# Profiling of Flavonoid and Antioxidant Activity of Fruit Tissues from 27 Chinese Local Citrus Cultivars

**DOI:** 10.3390/plants9020196

**Published:** 2020-02-05

**Authors:** Qiyang Chen, Dan Wang, Chun Tan, Yan Hu, Balasubramani Sundararajan, Zhiqin Zhou

**Affiliations:** 1 College of Horticulture and Landscape Architecture, Southwest University, Chongqing 400716, China; cqy0909@email.swu.edu.cn (Q.C.); wander007@email.swu.edu.cn (D.W.); tc870078395@163.com (C.T.); huyanswu@163.com (Y.H.); sundararajanbotany@gmail.com (B.S.); 2 The Southwest Institute of Fruits Nutrition, Liang jiang New District, Chongqing 401121, China; 3 Key Laboratory of Horticulture Science for Southern Mountainous Regions, Ministry of Education, Chongqing 400715, China

**Keywords:** local citrus cultivars, antioxidant activity, flavonoids, total phenolic

## Abstract

Flavonoid profile and antioxidant activity of citrus peels, pulps, and juices from 27 local citrus cultivars in China were investigated. Flavonoid composition and content were determined using UPLC-PDA. Total phenolic content (TPC) and total flavonoid content (TFC) were measured using a Folin–Ciocalteau reagent and Al(NO_3_)-NaNO_2_ complexometry, respectively. The antioxidant capacities of the extracts were evaluated by DPPH, ABTS and FRAP method, respectively. Citrus peel not only exhibited better antioxidant potential, but also presented more composition diversity and contained higher concentrations of flavonoids than pulp and juice. Different citrus species were characterized by their individual predominant flavonoids, contributing largely to the antioxidant activity, such as mandarin was characterized by hesperidin, nobiletin and tangeretin, while pummelo and papeda were characterized by naringin. The peel of Guihuadinanfeng (*Citrus reticulata*) had the highest TPC of 23.46 mg equivalent gallic acid/g DW (dry weight) and TFC of 21.37 mg equivalent rutin/g DW. Shiyueju (*C. reticulata*) peel showed the highest antioxidant capacity based on the antioxidant potency composite (APC) analysis. Overall, mandarin (*C. reticulata*) fruits peel contained more TPC and TFC, exhibiting higher antioxidant capacities than other species, and were good natural sources of flavonoids and antioxidants.

## 1. Introduction

Citrus fruits, which are widely cultivated worldwide, are important commercial crops [1]. Citrus fruits are well-accepted by consumers owing to their pleasant flavors and abundant phytochemicals, such as vitamins A and C, mineral elements, carotenoids, and phenolics [2,3]. In recent years, *Citrus* fruits have been suggested to be a good source of dietary antioxidants and their role in the prevention and treatment of various diseases were widely studied [4]. The major phenolic compounds found in citrus are flavonoids, which are considered to be one of the most important sources of bioactive compounds [5,6].

The phenolic hydroxyl groups attached to the ring structure of the flavonoids and are known to be antioxidants by scavenging free radicals, inhibiting lipid oxidation, or chelating metal ions [7]. Moreover, flavonoids affect the quality of fruits in terms of appearance, taste and nutritional value [8], and also effectively regulate many human diseases by mediating antioxidant, inflammatory, and immunomodulatory effects [9]. Generally, flavonoids in citrus included three main classified subclasses: flavanones, flavone-glycosides, and polymethoxylated flavones (PMFs) [10]. The significant compound of citrus flavonoids fruits is presented, including hesperidin, nobiletin, naringin, and neohesperidin. One of the PMFs nobiletin was reported to effectively inhibit the LPS-induced TNF-α in human monocytes and exhibited the potential anti-inflammatory effect [11]. In a combination of the hesperidin, nobiletin, and tangeretin synergistically enhanced the anti-inflammatory activity [12]. Tangeretin and nobiletin could be useful in cytostatic anticancer agents by inhibiting the proliferation of human cancers without inducing cell death [13]. To date, various analytical methods have been performed for qualify and quantify flavonoids through multiple methods, such as high-performance liquid chromatography (HPLC), capillary electrophoresis (CE) [14], high-performance electrospray ionization mass spectrometry (HPLC-UV-ESI-MS) [15], and photodiode array (PDA) detectors [16]. However, UPLC-PDA had many advantages in speed, cost, and precision [17].

**Figure 1:**
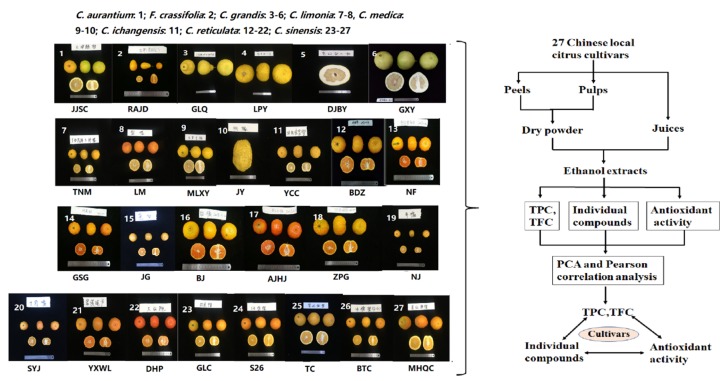
Workflow of this study. For abbreviation of cultivars see Section 4.2. TPC: total phenolic content; TFC: total flavonoid content.

China is one of the important origin centers of the genus *Citrus* L. with abundant cultivated and wild genotypes resources [18,19]. The *Citrus* genus has four basic species: *Citrus reticulata* Blanco (mandarin), *Citrus medica* L. (citron), *Citrus maxima* Burm. Merr (pummelo), and *Citrus micrantha* Wester (papeda), and secondary species: *Citrus sinensis* (L.) Osb. (sweet orange), *Citrus aurantifolia* L. (lime), *Citrus aurantium* L. (sour orange), *Citrus limon* (L.) Burm (lemon), and *Citrus paradisi* Macf (grapefruit) [20,21,22]. In recent years, *Citrus reticulata* has been described extensively in the literature [23]. However, *Citrus ichangensis* and *Citrus limonia* have been scarcely studied. Previous studies showed that the flavonoid types and contents in citrus fruits varied due to different species and cultivars [6], and their distribution in different fruits tissues [24]. Naringin was the dominant compound in the peel of pummelo (*C. grandis*), while mandarin (*C. reticulata*) was rich in hesperidin [25,26,27]. Grapefruits (Cocktail and Rio Red,) were more precious than the pummelo in flavonoids, and the contents of flavonoids in Cocktail (*C. paradisi*) peel was significantly higher than in its pulp. In contrast, the Changshanhuyou (*C. paradisi*) pulp was rich in nobiletin and tangeretin than the peel [25]. Considering these factors, citrus cultivars selected in our study was to analyze the flavonoids content, and composition of peels, pulps, and juices in 27 Chinese local citrus cultivars by UPLC-PDA, and their antioxidant capacities were evaluated (Figure 1). The principle component analysis (PCA) and Pearson correlation were applied to explore the relationship between flavonoid compounds and antioxidant capacity in different fruit tissues or different *Citrus* species. Our study contributes to finding new sources of active ingredients and lay the foundation for future utilization of local *Citrus* germplasm.

## 2. Results

### 2.1. TPC and TFC

The TPC showed significant differences (*p* < 0.05) among different cultivars, ranging from 3.31 to 23.46 mg GAE/g dry weight (DW) in peels, from 2.65 to 10.45 mg GAE/g DW in pulps, and from 0.12 to 1.37 mg GAE/mL fresh weight (FW) in juices (Table 1). Peels presented the highest TPC, followed by pulps, while juices had the lowest. As for peel, Guihuadinanfeng (NF) and Baiju (BJ) had significantly higher TPC than other cultivars tested, and these two cultivars belong to mandarin (*C. reticulata*).

Similarly, peels presented the highest TFC in most of the sample varied from 2.38 (Juyuan, JY, *C. medica*) to 21.37 (NF, *C. reticulata*) mg RE/g DW, compared with pulps and juices. Among all 27 citrus pulps studied, in particular, the highest value (16.42 mg RE/g DW) of TFC was observed in Ronganjindan (RAJD, *F. crassifolia*), whereas JY (*C. medica*) contained the lowest (1.89 mg RE/g DW). Gulaoqianshatianyou (GLQ, *C. grandis*) juices presented higher TFC (1.17 mg RE/mL FW) than those of other cultivars (Table 1).

### 2.2. Qualitative and Quantitative Analysis of Flavonoid Composition

A total of 18 major flavonoids, including six flavanones, one flavone, and 11 PMFs were identified from peels, pulps and juices based on retention time, characteristic spectrum comparison with the standards (Figure 2 and Figures S1–S76). And largely similar variation patterns of flavonoid components and contents were observed for the same citrus species studied (Figure 3, Figure S77 and Figure S78). Different species were characterized by different individual flavonoid compounds, such as pummelo was characterized by naringin, mandarin was characterized by hesperidin, nobiletin, and tangeretin (Table S1).

**Figure 4:**
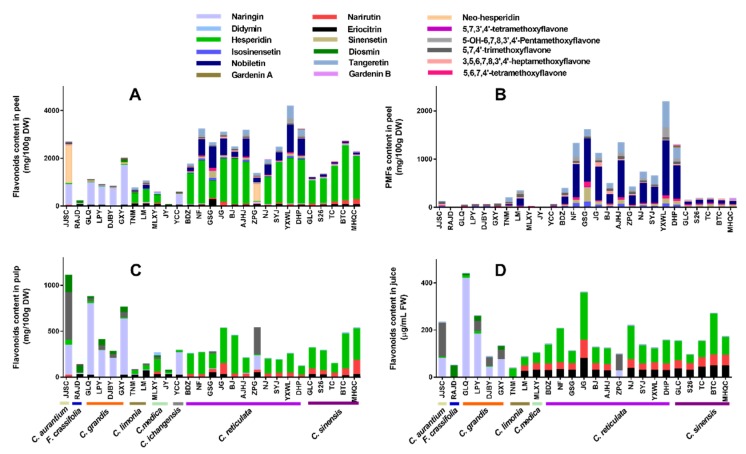
Flavonoids profile of different fruit parts from 27 citrus cultivars. (**A**) Flavonoids composition of peel. (**B**) PMFs of peel. (**C**) Flavonoids composition of pulp. (**D**) Flavonoids composition of juice.

The total flavanone content was observed in the range of 29.84–2554.70 mg/100 g DW in peels, 52.94–916.58 mg/100 g DW in pulps, and 28.30–392.48 μg/mL FW in juices (Figure 4 and Tables S2–S4). Among flavanones identified from the tested cultivars, hesperidin was the most dominant flavanone, followed by naringin and eriocitrin. The hesperidin contents varied from 0.00 to 2298.99 mg/100 g DW in peels, from 2.73 to 415.59 mg/100 g DW in pulps, from 0.00 to 199.35 μg/mL FW in juice, respectively. The hesperidin in different parts was largely as: peels > pulps > juices. Fourteen cultivars peels of *C. reticulata* and *C. sinensis* were the richest in hesperidin (960.15–2298.99 mg/100 g DW). Naringin contents varied from 0.00 to 1676.31 mg/100 g DW in peels, from 0.00 to 777.65 mg/100 g DW in pulps, from 0.00 to 419.28 μg/mL FW in juices, respectively. The naringin in different parts was largely as: peels > pulps > juices. *C. grandis* contained the highest level of naringin. Eriocitrin content varied from 0.00 to 268.69 mg/100 g DW in peels, from 0.00 to 66.72 mg/100 g DW in pulps, and from 0.00 to 78.39 μg/mL FW in juices, respectively. The eriocitrin in different parts was largely as: peels > pulps > juices. Cupigoushigan (GSG, *C. reticulata*) peel and Limeng (LM, *C. limonia*) contained the highest level of eriocitrin, while it was not detectable in *C. grandis*, *C. aurantium* and *F. crassifolia* juices. Jiangjinsuancheng (JJSC, *C. aurantium*) contained the highest neohesperidin (1620.77 mg/100 g DW for peel, 517.10 mg/100 g DW for pulp, 144.85 μg/mL FW for juice) followed by Wangcangzhoupigan (ZPG, *C. reticulata*). *C. sinensis* contain relatively high content of narirutin compared to other species. Rich diosmin (189.16 mg/100 g DW) and didymin (85.25 mg/100 g DW) were detected in JJSC (*C. aurantium*) pulp and Mulixiangyuan (MLXY, *C. medica*) peel.

PMFs are a unique type of flavonoids, mainly found in citrus peels compared to pulps and juices (Figure 4B and Table S5). The highest total of PMFs content (above 1000 mg/100 g DW) was found in 6 cultivars classified as *C. reticulata*: YXWL (2180.99 mg/100 g DW), GSG (1592.44 mg/100 g DW), Anjianghongju (AJHJ, 1323.01 mg/100 g DW), NF (1321.02 mg/g DW), Dahongpao (DHP, 1290.12 mg/g DW) and JG (1130.78 mg/g DW). Among the 11 PMFs identified, nobiletin, tangeretin, and sinensetin were three dominating PMFs in most citrus cultivars. YXWL (*C. reticulata*) contained the highest levels of nobiletin (1137.05 mg/100 g DW), tangeretin (536.75 mg/100 g DW), and 5-OH-6,7,8,3′,4′-pentamethoxyflavone (211.38 mg/100 g DW). Then GSG (*C. reticulata*) was found in the maximum amount of sinensetin (297.99 mg/100 g DW), isosinensetin (104.58 mg/100 g DW), and 5,6,7,4′-tetramethoxyflavone (105.40 mg/100 g DW). JG (*C. reticulata*) peel was rich in 3,5,6,7,8,3′,4′-heptamethoxyflavone (92.83 mg/100 g DW). It was remarkable that 18 citrus cultivars have been observed the similar content of 5,7,4′-trimethoxyflavone (31.57–60.75 mg/100 g DW) with a slight difference; however, *F. crassifolia*, *C. medica*, and *C. sinensis* were practically devoid of it. Rich Gardenin A (40.03 mg/100 g DW) was found in the peel of DHP, while Gardenin B was only observed in peels of three cultivars including DHP, Meihongqicheng (MHQC, *C. sinensis*), and Nianju (NJ, *C. reticulata*), it ranged from 11.87 to 27.89 mg/100 g DW.

### 2.3. Antioxidant Capacity

Antioxidant capacity of different fruit parts extracted from 27 citrus cultivars showed a significant variation using three methods which were 2,2′-azinobis 3-ethylbenzothiazoline-6-sulfonic acid (ABTS), 1,1-diphenyl-2-picrylhydrazyl radical (DPPH), and ferric reducing/antioxidant power (FRAP). The ABTS values of the cultivars tested varied from 25.07 to 461.72 μmol TE/g DW in peels, from 11.62 to 33.17 μmol TE/g DW in pulps, from 2.01 to 10.04 μmol TE/mL in juices, respectively (Table 2). The ABTS values in different tissues was largely as follows: peels > pulps > juices. NJ (*C. reticulata*) peel, Bingtangcheng (BTC, *C. sinensis*) pulp and GLQ (*C. grandis*) juice had significantly higher ABTS values than those in other cultivars. The DPPH level of the sample tested ranged from 21.80 to 357.18 μmol TE/g DW in peels, from 7.50 to 14.06 μmol TE/g DW in pulps, from 2.31 to 7.24 μmol TE/mL in juices, respectively. The highest DPPH radical ability was found in Shiyueju (SYJ, *C. reticulata*) peel, whereas the lowest DPPH radical ability was found in LM juice. As a whole, peels of all cultivars tested presented significantly higher DPPH radical ability than pulps and juices. *C. reticulata* peels presented relatively higher DPPH radical ability than other species peels. The FRAP values of cultivars tested varied from 6.90 to 39.77 μmol TE/g DW in peels, from 10.46 to 30.72 μmol TE/g DW in pulps, from 1.54 to 4.43 μmol TE/mL in juices. YXWL (*C. reticulata*) peel had the highest FRAP values, while RAJD (*F. crassifolia*) juice had the lowest FRAP value. For all tissues, peels of tested cultivars had higher FRAP values than other tissues, followed by pulps. The peels of mandarin had significantly higher FRAP values than other species tested, sour orange (*C. aurantium*) pulp had higher FRAP values than other cultivars tested.

ABTS, DPPH, and FRAP assay measured the antioxidant capacities of citrus extract based on different mechanisms, leading to different rank orders for the antioxidant capacity of the same cultivars. In the current study, an overall antioxidant potency composite index (APC index) was calculated, and the rank of different tissues of the citrus cultivars was shown in Table 2. The APC index of cultivars studied varied from 10.06% to 80.13% in peels, from 50.82% to 89.39% in pulps, from 31.70% to 100% in juices. The top six APC index in peels were classified as *C. reticulata*, of these cultivars, SYJ had the highest APC index. The antioxidant capacity of BTC (*C. sinensis*) pulp was higher than those from other citrus species pulps tested according to APC analysis. The GLQ and DJBY juice showed the highest antioxidant capacity, which both belong to the species of *C. grandis* (Table 2).

### 2.4. PCA Analysis

**Figure 5:**
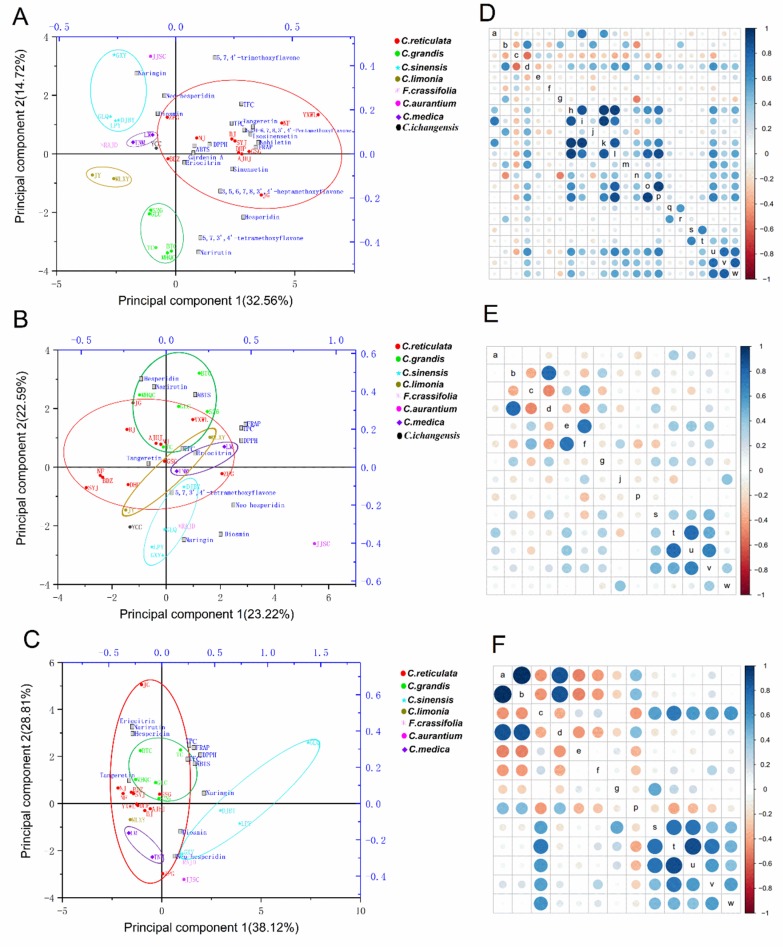
Biplot and Pearson correlation matrix for results obtained from peels (**A**,**D**), pulps (**B**,**E**), and juices (**C**,**F**) of 27 citrus cultivars. Different colored circle represented different citrus species (**A**–**C**). Figure inside letters are listed as: a-eriocitrin, b-narirutin, c-naringin, d-hesperidin, e-neohesperidin, f-diosmin, g-didymin, h-isosinensetin, i-sinensetin, j-5,7,3′,4′-tetramethoxyflavone, k-5,6,7,4′-tetramethoxyflavone, l-Nobiletin, m-3,5,6,7,8,3′,4′-heptamethoxyflavone, n-5,7,4′-trimethoxyflavone, o-5-hydroxy-6,7,8,3′,4′-pentamethoxyflavone, p-Tangeretin, q-gardenin A, r-gardenin B, s-ABTS, t-DPPH, u-FRAP, v-TPC, and w-TFC (**D**–**F**).

The PCA protocol was performed to standardize flavonoid contents, TFC, TPC, and antioxidant activity (ABTS, DPPH, and FRAP) obtained from 27 citrus cultivars. The first two principal components in citrus peels explained 47.28% of the total variability and clearly grouped citrus cultivars into eight different distinct clusters (Figure 5A). Principal component 1 (PC1) accounted for 32.56% of the total variance and the main dominant features were nobiletin, isosinensetin, tangeretin, and 5,6,7,4′-tetrathoxyflavone, while principal component 2 (PC2) represents 14.72% of the total variation and it was primarily dominated by narirutin, 5,7,3′,4′-tetramethoxyflavone, and hesperidin. The first two principal components in citrus pulps and juices explained 45.81% and 66.93% of the total variability, respectively (Figure 5B,C). Diosmin, naringin, neohesperidin, and TPC were the main predominant in pulps, and *C. reticulata*, *C. sinensis*, and *C. grandis* segment were roughly separated. Additionally, JJSC (*C. aurantium*) was separated by these compounds, as observed by the high correlation with PC2 (Figure 5B). In juices at mainly eriocitrin, narirutin, hesperidin, and TPC features of the JJSC (*C. aurantium*) and ZPG (*C. reticulata*) were showed the clustered together due to the neohesperidin and JG (*C. reticulata*) located at the farthest point according to the amount of the hesperidin (Figure 5C).

It was found that the FRAP test better expresses the antioxidant capacity of *Citrus*. Based on the resulting Pearson correlation values it can be stated that the factors having the highest influence on antioxidant activity in the FRAP test are PMFs, especially tangeretin (0.70, *p* < 0.01) with nobiletin (0.68, *p* < 0.01), as well as TPC (0.77, *p* < 0.01) and TFC (0.76, *p* < 0.01) in peels. In addition, FRAP had a moderate mutual correlation with 5,7,4′-trimethoxyflavone (0.44, *p* < 0.05) and sinensetin (0.43, *p* < 0.05) (Figure 5D). A similar correlation (0.68, *p* < 0.01) of the FRAP test and TPC was obtained in pulps (Figure 5E), while a strong correlation (0.74, *p* < 0.01) was observed between DPPH and TPC in juices (Figure 5F).

## 3. Discussion

To our knowledge, this is the first systematic report of flavonoids compositions and antioxidant capacity of peels, pulps, and juices of 27 Chinese local citrus cultivars. General, *Citrus* peels presented higher TPC and TFC than pulps and juices [28,29], which is consistent with our results. NF, BJ, JG, and SYJ contained the highest TPC (above 20 mg GAE/g DW). And these cultivars all belong to mandarin (*C. reticulata*). Zhang, et al. [30] reported TPC of the peels of 14 wild mandarin genotypes native to China, and the values range from 29.38 to 51.14 mg GAE/g DW. Our results were far below this range, and these differences probably ascribed the different extraction method details, region of production, cultivar and stage of ripeness. In another study, Chen et al. (2010) compared the extracts of 70% ethanol from seven regions of Zhejiang Province in China obtained a TPC for *C. reticulate* Blanco cv. Organ peel extracts from 15.60 to 19.00 mg GAE/g DW, which was lower than those of ours [31]. Previous results also investigated the TFC, and the content of Chinese wild mandarin varied from 7.95 to 20.66 mg RE/g DW in peels [30], and from 1.59 to 3.82 mg RE/g DW in pulps [32]. These data were similar to our results obtained from peels but lower than those of in pulps. This result suggested that the difference of TFC in the pulp between wild and local varieties may be caused by long-term domestication of citrus. TFC in peels were higher than pulps had been proved in previous studies [6,29]. In the current study, pulps of lemon (*C. limonia*) and kumquat (*F. crassifolia*) appeared a relatively higher TFC than those of in their peels (Table 1). These differences mainly explained by several factors such as genetic backgrounds, climate, cultivation system, and tree age [33,34,35].

Flavonoids are mainly present in *Citrus* fruits in the form of their glycosyl derivatives. Flavonoids composition and content in different tissues presented a significant variation among 27 Citrus cultivars. However, largely similar flavonoids variation pattern in the same citrus species provides direct evidence that flavonoid compounds are genetically controlled, which was similar to the previous result obtained by our group for the wild mandarin fruit [30]. Generally, citrus peels had more abundant flavonoids and higher contents than pulps and juices [29], which is consistent with our results. PMFs without the sugar moieties, appears less frequently in fruit pulps and juices, due to their lipophilicity and thus their low solubility in water [16]. In this study, three types of flavonoids, including flavanones, flavone, and PMFs, were detected and, of these, small amounts and types of PMFs were found in pulps and juices.

Flavanones, including hesperetin, naringenin, and their respective glycosides, are in the majority of flavonoids in *Citrus* species [36]. Previous result showed that sweet orange, Chinese wild mandarin, and lime were characterized by the highest level of hesperidin [30,36]. These studies were in line with our results that hesperidin was the principal flavanone in mandarin, lime, and sweet orange. Naringin and neohesperidin are rich in sour orange [37], and they are also the predominant flavanones in grapefruit. In the current study, local cultivar ZPG (*C. reticulata*), which might originate from interspecific hybridization between *C. sinensis* and *C. reticulata* [38], was characterized by neohesperidin and naringin. However, flavonoids are highly similar in the peels of JJSC (*C. aurantium*) and JPG (*C. reticulata*), which may correlate with high chemotype similarities between hybrids and their ascendants [39]. This result provides a new insight to *Citrus* classification and need further discussion on hybridization and genetic change. Pummelo (*C. grandis*) had a distinct flavanone characterization compared with grapefruit, was characterized by naringin [25,40], and our study also confirmed this result. Kumquat has four major genetic types, including *Fortunella japonica*, *Fortunella margarita*, *Fortunella crassifolia*, and *Fortunella hindsii*, and are rich in narirutin, hesperidin, and quercetin [41]. In our study, diosmin was the predominant flavone in *Fortunella crassifolia*. These results revealed *Citrus* species present their unique flavonoids profile.

PMFs are another unique type of flavonoids and mainly existed in *Citrus* peels. Previous study showed that isosinensetin, sinensetin, nobiletin, and tetramethyl-o-scutellarein (5,6,7,4′-tetramethoxyflavone) were the major PMFs in peels of *C. reticulata* Blanco cv. Ponkan [42]. Nobiletin proved to be the most dominant PMF in sweet orange and mandarin [6], and it ranged from 150.35 to 1137.05 mg/100 g DW in the present study, which was significantly higher than the content reported by Xing, et al. [43]. Meanwhile, mandarin peels were also the good source of tangeretin and senensetin. Tangeretin and nobiletin were the principal PMFs in *C. paradise* cv. Huyou [44], and tangeretin was the predominant PMF in *C. grandis* cv. Foyou. Gardenin A and gardenin B were only detected in peels of mandarin and sweet orange, which was in line with report by Xing et al. [43].

Citrus fruits have good antioxidant activities, due to the high content of flavonoids. DPPH, ABTS, and FRAP were three routine methods to evaluate the antioxidant capacity of plant extract [45,46,47]. *Citrus* peels showed the highest antioxidant capacity, followed by pulps, which is consistent with the content of individual compounds, TFC and TPC, similar with the ranking of the phytochemical content and antioxidant capacity in peels, pulps, and juices of citrus species and cultivars observed by Xi et al. [29] and Nogata et al. [48], showing that flavonoids may exhibit an important role in citrus antioxidant capacity. In the current study, except mandarin and *C. ichangensis*, *Citrus* pulps presented higher FRAP activity than peels, which may be owing to the composition of individual flavonoids and even their complicated interaction [29]. It can be found that FRAP better expresses the antioxidant capacity of citrus fruit than ABTS and DPPH, due to its high mutual correlation with the main component. As for species and cultivars, mandarin, such as NF, JG, and SYJ, not only contained higher TPC and TFC, but also had higher antioxidant capacities than other species and cultivars. At the same time, significantly higher flavonoids, including hesperidin, nobiletin, and tangeretin, were found in mandarin than those in other species tested, which was consistent with the results of antioxidant capacities, indicating that these compounds may play a key role in elevated antioxidant capacities. Some outstanding performances in our tests were found that antioxidant capacity measured by ABTS tested was higher than those of citrus reported by Xi et al. [29]. These results indicated Chinese local citrus cultivars or their different fruit parts have excellent antioxidant capacity, and should be utilized comprehensively.

## 4. Materials and Methods

### 4.1. Standards and Chemicals

Eriocitrin, narirutin, naringin, hesperidin, neo-hesperidin, diosmin, didymin 3,5,6,7,8,3′,4′-heptamethoxyflavone, Tangeretin, 5-hydroxy-6,7,8,3′,4′-pentamethoxyflavone, Gardenin A, Gardenin B were purchased from ChromaDex (ChromaDex Inc, Santa Ana, CA, USA). Isosinensetin, sinensetin, 5,7,3′,4′-tetramethoxyflavone, 5,6,7,4′-tetramethoxyflavone, 5,7,4′-trimethoxyflavone, and nobiletin were acquired from SinoStandards (Chengdu, China). Methanol (HPLC-grade) and formic acid were obtained from Sigma (St. Louis, MO, USA). 1,1-Diphenyl-2-picrylhydrazyl radical (DPPH), 2,2′-azinobis 3-ethylbenzothiazoline-6-sulfonic acid (ABTS), 6-hydroxy-2,5,7,8-tetramethylchroman-2-carboxylic acid (Trolox), and 2,4,6-tris (2-pyridyl)-S-triazine (TPTZ), dimethyl sulfoxide (DMSO) were purchased from Sigma (St. Louis, MO, USA). Deionized water (18.20 MΩ cm) was made with a Milli-Q system (Millipore, Bedford, MA, USA). All the other analytical grade reagents were obtained from Chuandong Chemical Reagent Co., Ltd. (Chongqing, China).

### 4.2. Plant Materials

Fruits of 27 citrus cultivars represent eight *Citrus* species, *Citrus aurantium* L., *Fortunella crassifolia* Swing., *C. grandis* Osbeck, *C. limonia* Osbeck, *C. medica* L., *C. ichangensis* Swing., *C. reticulata* Blanco, and *C. sinensis* Osbeck respectively, were sampled in this study. These 27 citrus cultivars were collected from 10 key production areas in China, including Jiangsu Province, Jiangxi Province, Sichuan Province, Chongqing City, Guangxi Province, Fujian Province, Shandong Province, Yunnan Province, Zhejiang Province, and Hunan Province. The information in detail for each sample is presented in Table 3. Based on their size and color, 30 sample fruits on the different parts of the crown were randomly collected from five fruit trees and divided into three groups as three replicates. All fruit samples were washed using water and manually separated into peels and pulps. Fresh pulps were squeezed into juices and store at −80 °C until analysis. Peels and pulps were placed in a 40 °C oven for 48 h and then dried materials were grounded into fine powder and sifted by a 60-mesh sieve for further use.

### 4.3. Sample Preparation

The extraction of flavonoids was performed, as described in the previous study [6]. Briefly, sample (peel 0.40 g, pulp 0.40 g, or juice 2 mL) was put into a 10 mL centrifuge tube and extracted with 8 mL anhydrous ethanol for 30 min in ultrasound (300 W) at room temperature. Then the extracts were centrifuged at 5000 rpm for 15 min. This extraction procedure was repeated three times and the supernatants were combined to a final volume of 25 mL in a brown volumetric flask. Extracts obtained from peel, pulp, and juice were used for determination of TPC, TFC, and antioxidant capacity. Extract (1 mL) was pipetted and filtered through 0.22 μm filter before UPLC-PDA analyses. Each sample has three independent repeats.

### 4.4. Determination of TPC and TFC

TPC was determined using the Folin–Ciocalteu method with minor modification [49]. Briefly, the above extract (0.30 mL) was add into 0.40 mL of Folin–Ciocalteu reagent. After 5 min of darkness, 2 mL of 5% Na_2_CO_3_ was added and the final volume was adjusted to 10 mL with distilled water. After incubating for 60 min at room temperature, the absorbance value was read at 765 nm. TFC was determined according to the method described by Kim [50] 0.20 mL of 5% NaNO_2_ and 0.70 mL distilled water were added to a 0.50 mL extract in a volumetric flask, and the mixture was kept for 6 min at room temperature. 0.30 mL of 10% Al(NO_3_)_3_ was added to the mixture and incubated for 6 min again, then 2 mL of 1 M NaOH was added. After incubating for 15 min at room temperature, the absorbance was measured at 500 nm. Results were expressed as mg of gallic acid equivalent (GAE) per gram of dry weight (DW) (mg GAE/g DW) of fruits for TPC, and mg of rutin equivalents (RE) per gram of DW (mg RE/g DW) for TFC.

### 4.5. UPLC-PDA Analysis

The UPLC-PDA analysis was performed on an ACQUITY UPLC instrument equipping with a PDA detector (Wates, Milford, MA, USA). Samples were separated by an ACQUITY UPLC BEH C18 column (2.10 × 100 mm, 1.70 mm, U.K.) at a column temperature of 40 °C. The parameters of the analytical method were performed according to our previous study [6]. The mobile phases consisted of solvent A (water with 0.01% formic acid) and solvent B (methanol), with the following gradient elution: 0–0.6 min, 10%–20% B; 0.6–5 min, 20%–70% B; 5–7 min, 70%–90% B; 7–9 min, 90%–10% B. The flow rate was 0.4 mL/min, and the detection peak is 330 nm for polymethoxylated flavones (PMFs), the detection peak is 283 nm for flavonoids (the PDA detector set range was 200–400 nm).

### 4.6. UPLC-PDA Method Validation

Calibration equations for quantified flavonoids were calculated at nine gradient concentrations. The detection limit (LOD) and the limit of quantitation (LOQ) were obtained based on the basic signal-to-noise ratio (S/N ≥ 3 or S/N ≥ 10). The inter- (three successive days) and intraday (one day) precision were studied by analyzing a same sample. Recovery was calculated according to the spiked concentration. The linear coefficient of variation of the flavonoid linear regression equation ranged from 0.9992 to 1.0000 with good linearity which presented in Table S6. The LOD and LOQ were less than 0.64 and 1.59 μg/mL, respectively. The intra-day (1.68–4.56%), inter-day precision (0.74–2.65%) and the spiked recovery (94.54–105.01%) indicate that the UPLC-PDA method for quantifying flavonoids is precise and trustworthy.

### 4.7. Assay of Antioxidant Capacity

ABTS, DPPH, and FRAP assays were conducted by the previous method [51]. For ABTS assay, 5 mL aqueous ABTS solution (7 mM) was added to 88 μL of 140 mM of a potassium per sulfate solution. The mixture was kept in dark at room temperature for 12–16 h before being used, and then diluted with ethanol to adjust the absorbance at 734 nm to 0.70 ± 0.02. Fruit extracts (40 μL) and reference substances Trolox, were allowed to react with 3.90 mL of the ABTS radical solution under dark conditions for 10 min. The absorbance at 734 nm was measured by microplate spectrophotometer (Varioskan Flash, Thermo Scientific, Waltham, MA, USA). For DPPH assay, 3.50 mL of DPPH was added into 500 μL ethanol extracts. After 30 min for darkness, the absorbance was detected at wavelength of 517 nm. FRAP reagent was prepared by mixing 200 mL acetate buffer (300 mM, pH 3.60), 20 mL TPTZ solution (10 mM in 40 mM HCl) and 20 mL of FeCl_3_·6H_2_O solution (20 mM). FRAP reagent (3.80 mL) was added to 200 μL of fruit extract. After 30 min at ambient temperature, the absorbance was detected at a wavelength of 593 nm. The results of ABTS, DPPH, and FRAP were represented as μmol of trolox equivalent (TE) per gram dry wight (μmol TE/g DW) for peels and pulps, and as μmol of trolox equivalent (TE) per milliliter fresh weight (μmol TE/mL FW) for juices, obtained from a trolox solution having reducing power equivalent to that of sample. Three repetitions were performed for the same sample.

### 4.8. Statistical Analysis

Statistical analysis was performed using SPSS 19.0 software (SPSS Inc., Chicago, IL, USA) and significant differences among the samples were calculated using one-way ANOVA followed by Duncan’s multiple-range test at the 5% level (*p* < 0.05). Data were expressed as the means ± SD (standard deviation). The antioxidant activity of citrus fruits was evaluated by APC index [52]. The APC index was calculated by assigning all assays an equal weight, assigning an index value of 100 to the best score for each test, and then calculating an index score for all other samples within the test as follows: antioxidant index score) [(sample score/best score) × 100]; the average of all three tests for each fruit part was then taken for the antioxidant potency composite index. PCA and Pearson correlation matrix was performed using Origin 2017 and the statistical program R (version 3.5.0), respectively.

## 5. Conclusions

Hesperidin and naringin were the most dominant flavanones in citrus tested, ranging from 0.00 to 2298.99 mg/100 g DW and 0.00 to 1676.31 mg/100 g DW, BTC (*C. sinensis*) and GXY (*C. medica*) peels with the highest contents, respectively. JJSC (*C. aurantium*) presented the highest level of neohesperidin and also a good source of naringin. Nobiletin was the predominant PMF, varying 0.00–1137.05 mg/100 g DW, YXWL (*C. reticulata*) contained the highest level of nobiletin. GSG (*C. reticulata*) peel contained rich sinensetin and isosinensetin. YXWL (*C. reticulata*) peel presented significant higher 5-OH-6,7,8,3′,4′-pentamethoxyflavone than other cultivars. Taken together, different species were characterized by their individual dominant flavonoids, cultivars from mandarin contained significantly higher flavonoid, also exhibited higher antioxidant capacities than cultivars from other species tested. Pummelo and papeda were characterized by naringin, while sour orange showed both naringin-rich and neohesperidin-rich species. Mandarin was characterized by hesperidin, nobiletin and tangeretin, and diosmin was the most predominant compound in kumquat. Further, PCA and Pearson correlation revealed the characteristic flavonoids for each species contributed largely to the antioxidant capacity. The order of flavonoid contents and antioxidant capacities for different fruit part was largely as: peels > pulps > juices. These findings provide a comprehensive flavonoid profile of 27 Chinese local citrus cultivars and useful information for utilization of local citrus germplasm.

## Figures and Tables

**Figure 2:**
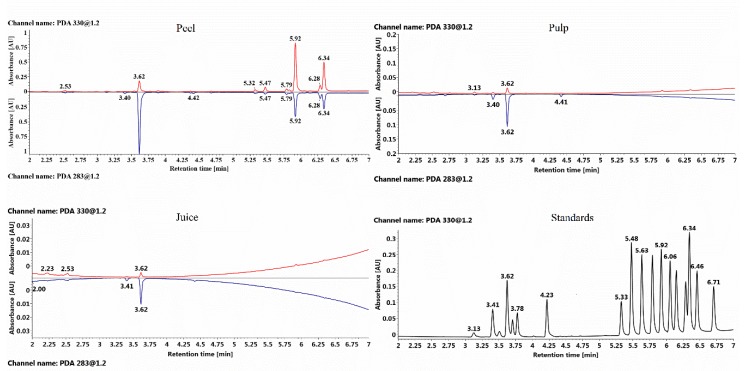
Characteristic UPLC-PDA chromatogram of YXWL (*C. reticulata*) peel, pulp, juice, and 18 flavonoid standards. Red lines and blue lines mean the chromatogram at the wavelengths of 330 nm and 283 nm, respectively. Retention time and corresponding compounds are listed as: 3.13 min, eriocitrin; 3.41 min, narirutin; 3.51 min, naringin; 3.62 min, hesperidin; 3.70 min, neohesperidin; 3.78, diosmin; 4.23 min, didymin; 5.33 min, isosinensetin; 5.48 min, sinensetin; 5.63 min, 5,7,3′,4′-tetrathoxyflavone; 5.79 min, 5,6,7,4′-tetrathoxyflavone; 5.92 min, nobiletin; 6.06 min, 3,5,6,7,8,3′,4′-hetamethoxyflavone; 6.15 min, 5,7,4′-trimethoxyflavone; 6.29 min, 5-hydroxy-6,7,8,3′,4′-pentamethoxyflavone; 6.34 min, tangeretin. 6.46, Gardenin A; 6.71 min, Gardenin B.

**Figure 3:**
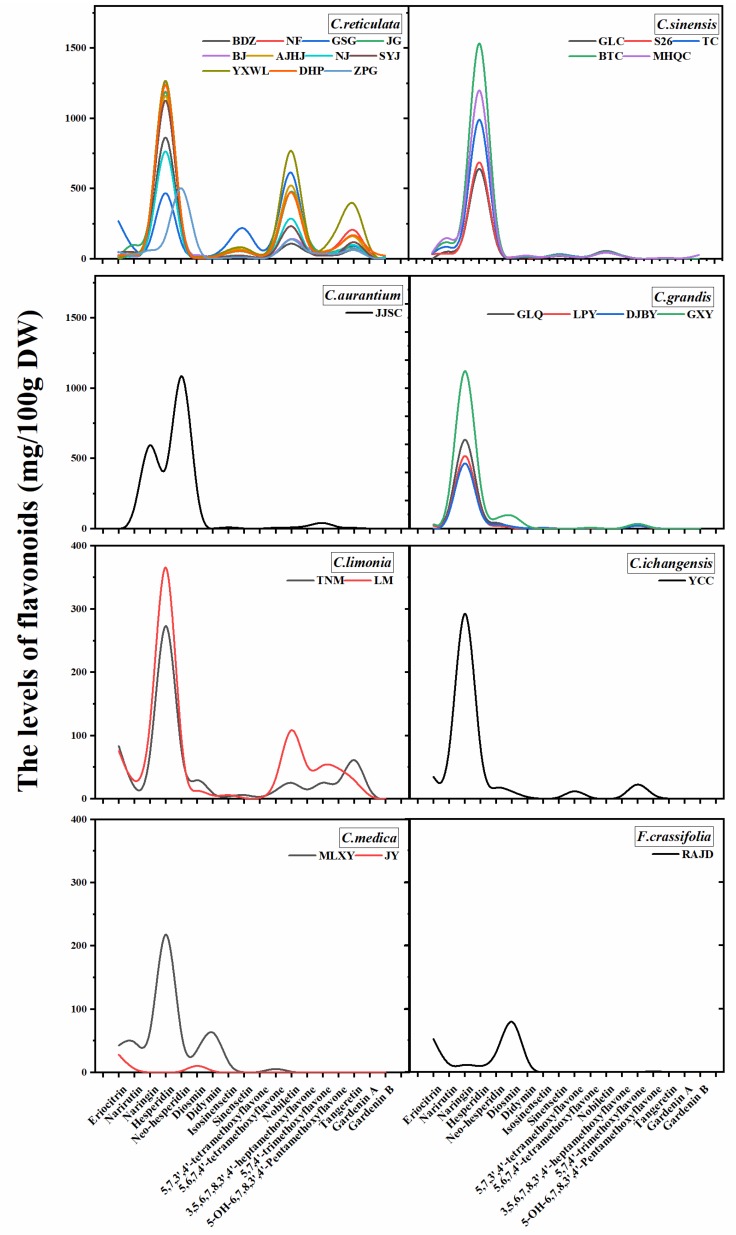
Variations pattern of flavonoids components and contents in the peel extracts of the 27 local cultivars (eight species) analyzed in this study.

**Table 1:** TPC and TFC in the different fruit tissues of 27 citrus cultivars.

No.	Cultivars	Peels		Pulps		Juices	
		TPC (mg/g GAE DW)	TFC (mg/g RE DW)	TPC (mg/g GAE DW)	TFC (mg/g RE DW)	TPC (mg/mL GAE)	TFC (mg/mL RE)
1	JJSC	18.15 ± 0.47 ^c^	17.09 ± 0.48 ^cd^	10.45 ± 0.29 ^a^	7.14 ± 0.12 ^ghi^	0.58± 0.043 ^def^	0.43 ± 0.01 ^gh^
2	RAJD	8.69 ± 0.49 ^k^	8.23 ± 0.27 ^kl^	7.33 ± 0.21 ^defg^	13.94 ± 0.06 ^b^	0.79 ± 0.05 ^c^	0.57 ± 0.03 ^de^
3	GXY	14.93 ± 0.21 ^d^	14.03 ± 0.46 ^fg^	7.02 ± 0.14 ^defg^	7.83 ± 0.43 ^fgh^	0.49 ± 0.02 ^fghi^	0.35 ± 0.03 ^ij^
4	DJBY	12.82 ± 0.46 ^e^	8.25 ± 0.28 ^kl^	7.19 ± 0.58 ^defg^	5.89 ± 0.31 ^ij^	0.63 ± 0.01 ^de^	0.54 ± 0.01 ^ef^
5	GLQ	10.62 ± 0.42 ^fghi^	9.30 ± 0.20 ^jk^	7.65 ± 0.43 ^cdef^	8.25 ± 0.42 ^efg^	1.27 ± 0.04 ^b^	1.17 ± 0.03 ^a^
6	LPY	8.79 ± 0.16 ^jk^	10.03 ± 0.07 ^j^	4.52 ± 0.75 ^ij^	3.67 ± 0.41 ^k^	0.81 ± 0.04 ^c^	0.48 ± 0.02 ^fg^
7	LM	9.48 ± 0.50 ^hijk^	6.17 ± 0.06 ^no^	7.09 ± 0.58 ^defg^	7.02 ± 0.11 ^ghi^	0.12 ± 0.01 ^k^	0.29 ± 0.02 ^j^
8	TNM	9.27 ± 0.58 ^ijk^	6.29 ± 0.09 ^mn^	6.47 ± 0.08 ^fgh^	16.42 ± 0.88 ^a^	0.22 ± 0.01 ^j^	0.55 ± 0.01 ^ef^
9	MLXY	8.88 ± 0.34 ^jk^	4.79 ± 0.25 ^o^	8.07 ± 0.47 ^bcdef^	5.08 ± 0.23 ^j^	0.42 ± 0.03 ^hi^	0.30 ± 0.04 ^j^
10	JY	3.31 ± 0.11 ^l^	2.38 ± 0.15 ^p^	3.89 ± 0.15 ^jk^	1.89 ± 0.08 ^l^	-	-
11	YCC	17.76 ± 1.17 ^c^	14.24 ± 0.54 ^fg^	5.08 ± 0.16 ^hij^	5.47 ± 0.18 ^j^	-	-
12	NF	23.46 ± 1.19 ^a^	21.37 ± 0.74 ^a^	4.41 ± 0.41 ^ij^	3.88 ± 0.24 ^k^	0.53 ± 0.01 ^efg^	0.33 ± 0.01 ^ij^
13	BJ	22.59 ± 0.91 ^a^	20.16 ± 0.49 ^ab^	6.88 ± 0.17 ^efg^	7.52 ± 0.35 ^gh^	0.42 ± 0.02 ^hi^	0.37 ± 0.01 ^hi^
14	SYJ	20.66 ± 0.83 ^b^	16.38 ± 1.09 ^d^	2.65 ± 0.32 ^k^	5.43 ± 0.73 ^j^	0.43 ± 0.02 ^ghi^	0.39 ± 0.03 ^hi^
15	JG	20.42 ± 0.32 ^b^	15.79 ± 0.09 ^de^	7.31 ± 0.47 ^defg^	9.66 ± 0.36 ^d^	1.37 ± 0.08 ^a^	0.68 ± 0.03 ^c^
16	AJHJ	15.65 ± 0.31 ^d^	9.61 ± 0.39 ^jk^	7.17 ± 0.54 ^defg^	6.87 ± 0.22 ^hi^	0.30 ± 0.01 ^j^	0.19 ± 0.01 ^k^
17	YXWL	14.79 ± 0.41 ^d^	19.77 ± 0.51 ^b^	9.12 ± 0.59 ^abc^	7.21 ± 0.77 ^ghi^	0.49 ± 0.03 ^fghi^	0.39 ± 0.03 ^hi^
18	ZPG	12.74 ± 0.13 ^e^	12.66 ± 0.15 ^gh^	6.66 ± 1.03 ^fg^	8.24 ± 0.32 ^efg^	0.28 ± 0.01 ^j^	0.36 ± 0.02 ^hij^
19	NJ	12.22 ± 0.96 ^ef^	12.70 ± 0.60 ^gh^	8.55 ± 0.87 ^bcd^	9.31 ± 0.48 ^de^	0.39 ± 0.02 ^i^	0.51 ± 0.01 ^ef^
20	GSG	11.33 ± 0.53 ^efg^	12.94 ± 0.76 ^gh^	5.79 ± 0.31 ^ghi^	5.21 ± 0.50 ^j^	0.40 ± 0.01 ^i^	0.58 ± 0.04 ^de^
21	BDZ	11.14 ± 0.23 ^efgh^	7.57 ± 0.29 ^lm^	4.25 ± 0.22 ^ij^	10.90 ± 0.62 ^c^	0.43 ± 0.01 ^hi^	0.62 ± 0.03 ^cd^
22	DHP	10.58 ± 0.34 ^fghi^	14.94 ± 0.43 ^ef^	4.84 ± 0.05 ^ij^	7.27 ± 0.44 ^gh^	0.58 ± 0.02 ^def^	0.34 ± 0.02 ^ij^
23	GLC	12.68 ± 0.39 ^e^	5.92 ± 0.73 ^no^	8.43 ± 0.14 ^bcde^	9.81 ± 0.39 ^cd^	0.67 ± 0.03 ^d^	0.35 ± 0.02 ^ij^
24	MHQC	12.32 ± 0.21 ^ef^	11.62 ± 0.20 ^hi^	6.95 ± 0.32 ^defg^	7.15 ± 0.42 ^ghi^	0.58 ± 0.04 ^def^	0.38 ± 0.01 ^hi^
25	TC	11.73 ± 0.56 ^efg^	5.89 ± 0.43 ^no^	4.98 ± 0.06 ^ij^	5.32 ± 0.21 ^j^	0.67 ± 0.02 ^d^	0.43 ± 0.02 ^gh^
26	BTC	11.57 ± 0.32 ^efg^	10.66 ± 0.27 ^ij^	9.49 ± 1.07 ^ab^	9.26 ± 0.21 ^de^	0.47 ± 0.04 ^ghi^	0.92 ± 0.02 ^b^
27	S26	10.43 ± 0.45 ^ghij^	10.29 ± 0.31 ^ij^	9.21 ± 0.07 ^ab^	9.00 ± 0.15 ^def^	0.51 ± 0.01 ^fgh^	0.23 ± 0.01 ^k^

Data are expressed as means ± SD. The different small letters after the means represent significance in the same tissue of citrus at 0.05 level. -: not detected. For abbreviation see Section 4.2. *C. aurantium*: No.1, *F. crassifolia*: No.2, *C. grandis*: No.3–6, *C. limonia*: No.7–8, *C. medica*: No. 9–10, *C. ichangensis*: No.11, *C. reticulata*: No.12–22, *C. sinensis*: No.23–27.

**Table 2:** The antioxidant activities of different fruit tissues (peel, pulp (μmol/g TE DW), and juice (μmol/mL TE) were evaluated by ABTS, DPPH, and FRAP methods.

NO.	Cultivars	Peels					Pulps					Juices				
		ABTS	DPPH	FRAP	APC	Rank	ABTS	DPPH	FRAP	APC	Rank	ABTS	DPPH	FRAP	APC	Rank
1	JJSC	60.51 ± 1.96 ^gfh^	38.58 ± 0.69 ^hi^	19.20 ± 0.17 ^fg^	24.92	13	18.88 ± 1.30 ^hijk^	12.60 ± 0.11 ^b^	30.72 ± 1.55 ^a^	82.18	6	3.24 ± 0.13 ^ijkl^	2.88 ± 0.06 ^lm^	1.80 ± 0.03 ^ghi^	37.56	19
2	RAJD	46.48 ± 0.50 ^h^	26.39 ± 0.76 ^i^	6.90 ± 0.37 ^l^	11.91	25	19.33 ± 1.79 ^hij^	7.89 ± 0.12 ^hi^	19.81 ± 0.26 ^gh^	59.63	20	2.53 ± 0.16 ^lm^	3.48 ± 0.04 ^hij^	1.54 ± 0.18 ^i^	36.01	24
3	DJBY	53.25 ± 1.74 ^gh^	29.86 ± 2.07 ^hi^	9.96 ± 0.44 ^kl^	15.43	22	31.14 ± 1.49 ^a^	10.68 ± 0.35 ^cde^	17.97 ± 1.28 ^hij^	76.11	10	9.61 ± 0.43 ^a^	5.30 ± 0.04 ^c^	3.17 ± 0.21 ^c^	80.16	2
4	GXY	68.54 ± 2.24 ^gf^	29.57 ± 1.52 ^hi^	8.45 ± 0.15 ^l^	15.17	23	16.15 ± 0.34 ^jk^	8.25 ± 0.17 ^ghi^	14.60 ± 0.69 ^klm^	51.63	25	3.24 ± 0.20 ^ijkl^	4.06 ± 0.05 ^e^	2.22 ± 0.20 ^defgh^	46.15	12
5	LPY	39.77 ± 1.12 ^hi^	24.00 ± 0.75 ^i^	10.67 ± 0.30 ^kl^	14.53	24	15.59 ± 0.90 ^k^	9.39 ± 0.31 ^fg^	18.59 ± 0.59 ^hi^	58.10	21	5.02 ± 0.17 ^de^	5.77 ± 0.09 ^b^	3.27 ± 0.30 ^c^	67.84	4
6	GLQ	47.22 ± 2.18 ^h^	20.90 ± 0.71 ^i^	7.13 ± 0.27 ^l^	11.66	26	25.18 ± 0.73 ^bcde^	9.12 ± 0.86 ^fgh^	12.64 ± 0.35 ^n^	60.64	18	10.04 ± 0.50 ^a^	7.24 ± 0.28 ^a^	4.43 ± 0.54 ^a^	100.00	1
7	LM	52.66 ± 3.59 ^gh^	33.75 ± 2.97 ^hi^	16.47 ± 2.81 ^ghij^	21.49	17	26.34 ± 0.58 ^bc^	14.06 ± 0.18 ^a^	24.98 ± 0.41 ^def^	86.91	2	2.89 ± 0.17 ^jklm^	2.31 ± 0.09 ^n^	2.24 ± 0.13 ^defgh^	37.09	20
8	TNM	50.93 ± 1.78 ^gh^	30.06 ± 0.53 ^hi^	14.68 ± 0.42 ^hij^	19.44	21	21.34 ± 1.63 ^fghi^	10.55 ± 0.14 ^de^	21.04 ± 1.65 ^gh^	69.29	13	2.01 ± 0.54 ^m^	2.38 ± 0.15 ^n^	1.87 ± 0.15 ^fghi^	31.70	25
9	MLXY	142.83 ± 4.12 ^c^	87.94 ± 5.27 ^d^	6.68 ± 0.28 ^l^	24.42	14	26.88 ± 1.74 ^bc^	12.66 ± 0.34 ^b^	26.31 ± 1.75 ^cde^	85.57	3	3.34 ± 0.01 ^hijkl^	2.79 ± 0.08 ^m^	1.72 ± 0.02 ^hi^	36.88	21
10	JY	25.07 ± 1.72 ^i^	21.8 ± 2.08 ^i^	7.04 ± 0.47 ^l^	10.06	27	20.98 ± 1.58 ^ghi^	8.65 ± 0.41 ^hi^	15.05 ± 1.14 ^jkl^	57.92	22	-	-	-	-	-
11	YCC	156.76 ± 5.14 ^bc^	42.12 ± 3.23 ^hi^	24.65 ± 1.12 ^d^	35.91	7	11.62 ± 0.37^l^	9.30 ± 0.31 ^fg^	16.68 ± 0.25 ^ijk^	51.82	24	-	-	-	-	-
12	SYJ	186.46 ± 14.49 ^b^	357.18 ± 21.76 ^a^	37.75 ± 0.10 ^ab^	80.13	1	21.58 ± 0.81 ^efgh^	7.50 ± 0.28 ^i^	10.46 ± 0.32 ^ef^	50.82	27	4.06 ± 0.14 ^efghi^	3.55 ± 0.05 ^ghi^	2.50 ± 0.16 ^de^	48.63	10
13	NJ	461.72 ± 21.34 ^a^	200.37 ± 14.21 ^b^	12.74 ± 1.05 ^jk^	63.28	2	23.49 ± 0.67 ^cdefg^	9.24 ± 0.33 ^fg^	22.60 ± 0.69 ^fg^	70.03	12	2.23 ± 0.20 ^m^	3.10 ± 0.03 ^kl^	1.92 ± 0.10 ^efghi^	36.12	23
14	NF	131.64 ± 13.14 ^c^	128.65 ± 7.02 ^c^	35.45 ± 0.92 ^bc^	52.81	3	20.92 ± 0.37 ^ghi^	8.95 ± 0.80 ^fgh^	10.58 ± 0.16 ^n^	53.72	23	4.64 ± 0.36 ^defg^	2.92 ± 0.05 ^lm^	1.58 ± 0.22 ^i^	40.74	17
15	BJ	94.15 ± 7.09 ^de^	69.75 ± 1.86 ^ef^	37.15 ± 3.64 ^abc^	46.11	4	24.98 ± 0.63 ^bcdef^	8.92 ± 0.24 ^gh^	15.44 ± 0.42 ^jkl^	63.00	16	4.13 ± 0.31 ^efghi^	3.60 ± 0.08 ^gh^	1.77 ± 0.03 ^ghi^	43.60	15
16	JG	110.74 ± 2.04 ^d^	66.04 ± 3.58 ^ef^	33.96 ± 1.46 ^c^	44.14	5	22.52 ± 1.03 ^defgh^	8.26 ± 0.19 ^ghi^	16.39 ± 0.62 ^ijk^	60.00	19	3.64 ± 0.09 ^ghijk^	4.69 ± 0.11 ^d^	3.14 ± 0.13 ^c^	57.30	5
17	YXWL	59.16 ± 2.20 ^gh^	48.42 ± 0.94 ^gh^	39.77 ± 1.03 ^a^	43.91	6	24.52 ± 1.80 ^bcdefg^	11.84 ± 0.23 ^bc^	29.49 ± 1.56 ^ab^	84.71	5	3.93 ± 0.50 ^fghi^	3.30 ± 0.08 ^ijk^	1.93 ± 0.18 ^efghi^	42.76	16
18	AJHJ	78.96 ± 6.03 ^ef^	79.42 ± 10.51 ^de^	23.37 ± 1.14 ^de^	33.75	8	26.75 ± 1.68 ^bc^	10.19 ± 0.35 ^ef^	20.70 ± 0.90 ^gh^	73.50	11	4.30 ± 0.19 ^efgh^	3.91 ± 0.08 ^ef^	2.77 ± 0.16 ^cd^	53.12	9
19	GSG	43.52 ± 1.99 ^hi^	36.81 ± 1.19 ^hi^	26.83 ± 1.37 ^d^	30.27	9	16.53 ± 0.69 ^jk^	10.80 ± 0.10 ^cde^	24.00 ± 0.12 ^lmn^	68.30	14	6.24 ± 0.45 ^c^	3.24 ± 0.01 ^jk^	2.75 ± 0.12 ^cd^	56.33	7
20	ZPG	44.45 ± 3.25 ^h^	38.48 ± 0.90 ^hi^	25.16 ± 0.84 ^de^	29.02	10	25.27 ± 1.93 ^bcde^	11.01 ± 0.61 ^cde^	27.63 ± 1.31 ^bcd^	81.48	7	2.70 ± 0.13 ^klm^	3.14 ± 0.10 ^kl^	1.76 ± 0.05 ^hi^	36.66	22
21	BDZ	47.01 ± 2.72 ^h^	39.28 ± 2.09 ^hi^	21.93 ± 1.06 ^ef^	26.42	12	17.65 ± 0.27 ^ijk^	8.57 ± 0.60 ^ghi^	11.87 ± 0.56 ^mn^	50.93	26	4.82 ± 0.65 ^def^	3.29 ± 0.03 ^ijk^	1.82 ± 0.09 ^ghi^	44.84	14
22	DHP	51.16 ± 2.33 ^gh^	32.55 ± 0.56 ^hi^	19.41 ± 0.69 ^fg^	23.87	15	17.71 ± 0.95 ^ijk^	9.01 ± 0.21 ^fgh^	20.12 ± 0.72 ^gh^	60.99	17	3.27 ± 0.07 ^hijkl^	3.30 ± 0.09 ^ijk^	1.76 ± 0.23 ^hi^	39.29	18
23	GLC	98.82 ± 2.81 ^d^	58.69 ± 4.02 ^fg^	18.53 ± 0.76 ^fgh^	28.97	11	31.72 ± 0.88 ^a^	9.02 ± 0.36 ^fgh^	24.22 ± 0.59 ^ef^	79.54	9	5.40 ± 0.16 ^cd^	4.15 ± 0.04 ^e^	2.45 ± 0.17 ^def^	55.47	8
24	BTC	60.17 ± 1.14 ^gf^	34.17 ± 2.39 ^hi^	17.29 ± 0.60 ^ghi^	22.80	16	33.17 ± 0.86 ^a^	10.83 ± 0.33 ^cde^	28.00 ± 1.10 ^abcd^	89.39	1	3.99 ± 0.31 ^fghi^	3.77 ± 0.04 ^fg^	2.37 ± 0.05 ^defg^	48.44	11
25	MHQC	55.30 ± 2.36 ^gh^	30.33 ± 0.69 ^hi^	16.37 ± 1.00 ^ghij^	21.28	18	22.31 ± 0.29 ^defgh^	9.13 ± 0.21 ^fgh^	18.83 ± 0.58 ^hi^	64.50	15	2.53 ± 0.23 ^lm^	4.03 ± 0.02 ^ef^	2.42 ± 0.05 ^def^	45.16	13
26	S26	46.88 ± 6.84 ^h^	29.67 ± 2.19 ^hi^	15.76 ± 1.28 ^ghj^	20.07	19	25.96 ± 0.43 ^bcd^	11.54 ± 0.34 ^bcd^	29.25 ± 0.89 ^abc^	85.19	4	3.85 ± 0.34 ^fghij^	4.51 ± 0.07 ^d^	3.09 ± 0.09 ^c^	56.80	6
27	TC	46.31 ± 2.09 ^h^	36.71 ± 0.91 ^hi^	14.49 ± 1.28 ^ij^	19.56	20	27.36 ± 1.41 ^b^	10.65 ± 0.32 ^cde^	26.04 ± 1.93 ^de^	81.00	8	7.62 ± 0.21 ^b^	5.32 ± 0.09 ^c^	3.87 ± 0.02 ^b^	78.91	3

Data are expressed as means ± SD. The different small letters after the means represent significance in the same tissue of citrus at 0.05 level. -: not detected. For abbreviation see Section 4.2. APC was expressed as %.

**Table 3:** The information of citrus materials used in this study.

No.	Cultivar Denomination	Classification	Locality (China)	Repository Number	Abbreviation
1	Jiangjinsuancheng	*C. aurantium* L.	Jiangjin District, Chongqing City	LA0002	JJSC
2	Ronganjindan	*F. crassifolia* Swing.	Rongan County, Guangxi Province	LF0006	RAJD
3	Gulaoqianshatianyou	*C. grandis* Osbeck	Changshou District, Chongqing City	LG0004	GLQ
4	Liangpingyou1hao	*C. grandis* Osbeck	Liangping County, Chongqing City	LG0006	LPY
5	Dianjiangbaiyou	*C. grandis* Osbeck	Dianjiang District, Chongqing City	LG0007	DJBY
6	Guanximiyou	*C. grandis* Osbeck	Pinghe County, Fujian Province	LG0038	GXY
7	Pingxiangtuningmeng	*C. limonia* Osbeck	Pingxiang City, Guangxi Province	LM0032	TNM
8	Limeng	*C. limonia* Osbeck	Junan County, Shandong Province	LM0030	LM
9	Mulixiangyuan	*C. medica* L.	Muli County, Sichuan Province	LM0097	MLXY
10	Juyuan	*C. medica* L.	Yunnan Province	LM0091	JY
11	Huaihuayichangcheng	*C. ichangensis*	Huaihua City, Hunan Province	LP0017	YCC
12	Bendizao	*C. reticulata* Blanco	Huangyan District, Zhejiang Province	LR0001	BDZ
13	Guihuadinanfeng	*C. reticulata* Blanco	Nanfeng County, Jiangxi Province	LR0010	NF
14	Cupigoushigan	*C. reticulata* Blanco	Hechuan District, Chongqing City	LR0018	GSG
15	Shantoujiaogan	*C. reticulata* Blanco	Puning City, Guangdong Province	LR0022	JG
16	Baiju	*C. reticulata* Blanco	Jianshui County, Yunnan Province	LR0027	BJ
17	Anjianghongju	*C. reticulata* Blanco	Qianyang County, Hunan Province	LR0036	AJHJ
18	Wangcangzhoupigan	*C. reticulata* Blanco	Wangcang County, Sichuan Province	LR0072	ZPG
19	Nianju	*C. reticulata* Blanco	Dafeng District, Jiangsu Province	LR0093	NJ
20	Shiyueju	*C. reticulata* Blanco	Longmen County, Guangdong Province	LR0310	SYJ
21	Yanxiwanlu	*C. reticulata* Blanco	Xinhui District, Guangdong Province	LR0361	YXWL
22	Dahongpao	*C. reticulata* Blanco	Meishan City, Sichuan Province	LR0094	DHP
23	Gailiangcheng	*C. sinensis* (L.) Osbeck	Fujian Province	LS0019	GLC
24	S26Jincheng	*C. sinensis* (L.) Osbeck	Youxi County, Fujian Province	LS0040	S26
25	Lixingtiancheng	*C. sinensis* (L.) Osbeck	Jiangjin District, Chongqing City	LS0055	TC
26	Bingtangcheng	*C. sinensis* (L.) Osbeck	Jiangjin District, Chongqing City	LS0101	BTC
27	Meihongqicheng	*C. sinensis* (L.) Osbeck.	Fengjie County, Chongqing City	LS0033	MHQC

## Supplementary Materials

The following are available online at https://www.mdpi.com/2223-7747/9/2/196/s1, Figure S1. The UV–VIS spectrum of the 18 flavonoids tested in this study, Figures S2–S76. Characteristic UPLC-PDA chromatogram of different cultivars from peel, pulp, and juice, Figures S77 and S78. Variations pattern of flavonoids components and contents in the peel and juice extracts of the 27 local cultivars analyzed in this study, Table S1. The characterized component in citrus peels from eight citrus species, Tables S2–S5. Flavonoids in the peels, pulps and juices of 27 citrus cultivars, Table S6. Linearity, LOD, LOQ, recoveries, and precision of 18 standard flavonoids obtained by UPLC-PDA.

## References

[1] Mahato N., Sharma K., Sinha M., Cho M.H. (2018). Citrus waste derived nutra-/pharmaceuticals for health benefits: Current trends and future perspectives. J. Funct. Foods.

[2] Zou Z., Xi W.P., Hu Y., Nie C., Zhou Z.Q. (2016). Antioxidant activity of *Citrus* fruits. Food Chem..

[3] Tadeo F.R., Cercós M., Colmenero-Flores J.M., Iglesias D.J., Naranjo M.A., Ríos G., Carrera E., Ruiz-Rivero O., Lliso I., Morillon R. (2008). Molecular physiology of development and quality of citrus. Adv. Bot. Res..

[4] Cirmi S., Navarra M., Woodside J.V., Cantwell M.M. (2018). Citrus fruits intake and oral cancer risk: A systematic review and meta-analysis. Pharm. Res..

[5] Liu F., Wang M., Wang M. (2018). Phenolic compounds and antioxidant activities of flowers, leaves and fruits of five crabapple cultivars (*Malus Mill.* species). Acta Horticuhurae Sin..

[6] Zhao Z.Y., He S.S., Hu Y., Yang Y., Jiao B.N., Fang Q., Zhou Z.Q. (2017). Fruit flavonoid variation between and within four cultivated *Citrus* species evaluated by UPLC-PDA system. Acta Horticuhurae Sin..

[7] Tripoli E., Guardia M.L., Giammanco S., Majo D.D., Giammanco M. (2007). Citrus flavonoids: Molecular structure, biological activity and nutritional properties: A review. Food Chem..

[8] Durand-Hulak M., Dugrand A., Duval T., Bidel L.P., Jay-Allemand C., Froelicher Y., Bourgaud F., Fanciullino A.L. (2015). Mapping the genetic and tissular diversity of 64 phenolic compounds in *Citrus* species using a UPLC-MS approach. Ann. Bot..

[9] Yi L., Ma S., Ren D. (2017). Phytochemistry and bioactivity of *Citrus* flavonoids: A focus on antioxidant, anti-inflammatory, anticancer and cardiovascular protection activities. Phytochem. Rev..

[10] Gao Z., Gao W., Zeng S.L., Li P., Liu E.H. (2018). Chemical structures, bioactivities and molecular mechanisms of citrus polymethoxyflavones. J. Funct. Foods.

[11] Manthey J.A., Grohmann K., Montanari A., Ash K., Manthey C.L. (1999). Polymethoxylated flavones derived from citrus suppress tumor necrosis factor-r expression by human monocyte. J. Nat. Prod..

[12] Ho S.C., Kuo C.T. (2014). Hesperidin, nobiletin, and tangeretin are collectively responsible for the anti-neuroinflammatory capacity of tangerine peel (*Citri reticulatae* pericarpium). Food Chem. Toxicol..

[13] Morley K.L., Ferguson P.J., Koropatnick J. (2007). Tangeretin and nobiletin induce G1 cell cycle arrest but not apoptosis in human breast and colon cancer cells. Cancer Lett..

[14] Singh B., Kumar A., Malik A.K. (2017). Flavonoids biosynthesis in plants and its further analysis by capillary electrophoresis. Electrophoresis.

[15] Brito A., Ramirez J.E., Areche C., Sepulveda B., Simirgiotis M.J. (2014). HPLC-UV-MS profiles of phenolic compounds and antioxidant activity of fruits from three citrus species consumed in Northern Chile. Molecules.

[16] Gattuso G., Barreca D., Gargiulli C., Leuzzi U., Caristi C. (2007). Flavonoid composition of *Citrus* juices. Molecules.

[17] Hu K., Deng Z., Li S., Wu M., Liu W., Zhang S. (2017). SPE-UHPLC-DAD method for the simultaneous determination of three flavonoids in grape juice by using bis(tetraoxacalix [2]arene[2]triazine)-modified silica as sorbent. Food Anal. Methods.

[18] Biswas M.K., Chai L.J., Amar M.H., Zhang X.L., Deng X.X. (2011). Comparative analysis of genetic diversity in *Citrus* germplasm collection using AFLP, SSAP, SAMPL and SSR markers. Acta Horticuhurae Sin..

[19] Hazarika T.K., Hazarika B.N., Shukla A.C. (2014). Genetic variability and phylogenetic relationships studies of genus *Citrus* L. with the application of molecular markers. Genet. Resour. Crop. Evol..

[20] Nicolosi E., Deng Z.N., Gentile A., Continella S.L.M.G., Tribulato E. (2000). Citrus phylogeny and genetic origin of important species as investigated by molecular markers. Theor. Appl. Gene.

[21] Froelicher Y., Mouhaya W., Bassene J.B., Costantino G., Kamiri M., Luro F., Morillon R., Ollitrault P. (2010). New universal mitochondrial PCR markers reveal new information on maternal citrus phylogeny. Tree Genet. Genomes.

[22] Ghada B., Amel O., Aymen M., Aymen A., Amel S.H. (2019). Phylogenetic patterns and molecular evolution among ‘True citrus fruit trees’ group (Rutaceae family and Aurantioideae subfamily). Acta Horticuhurae Sin..

[23] Ke Z.L., Yang Y., Tan S., Zhou Z.Q. (2017). Characterization of Polymethoxylated Flavonoids in the Peels of Chinese Wild Mandarin (*Citrus reticulata* Blanco) by UPLC-Q-TOF-MS_MS. Food Anal. Methods.

[24] Fu M.Q., Xu Y.J., Chen Y.L., Wu J.J., Yu Y.S., Zou B., An K.J., Xiao G.S. (2017). Evaluation of bioactive flavonoids and antioxidant activity in Pericarpium Citri Reticulatae (*Citrus reticulata* ‘Chachi’) during storage. Food Chem..

[25] Xi W.P., Fang B., Zhao Q.Y., Jiao B.N., Zhou Z.Q. (2014). Flavonoid composition and antioxidant activities of Chinese local pummelo (*Citrus grandis* Osbeck.) varieties. Food Chem..

[26] Zhang H., Yang Y.F., Zhou Z.Q. (2017). Phenolic and flavonoid contents of mandarin (*C. reticulata* Blanco) fruit tissues and their antioxidant capacity as evaluated by DPPH and ABTS methods. J. Integr. Agric..

[27] Zhang M.X., Duan C.Q., Zang Y.Y., Huang Z.W., Liu G.J. (2011). The flavonoid composition of flavedo and juice from the pummelo cultivar (*Citrus grandis* (L.) Osbeck) and the grapefruit cultivar (*Citrus paradisi*) from China. Food Chem..

[28] Dong X.Y., Hu Y., Li Y., Zhou Z.Q. (2019). The maturity degree, phenolic compounds and antioxidant activity of Eureka lemon [*Citrus limon* (L.) Burm. f.]: A negative correlation between total phenolic content, antioxidant capacity and soluble solid content. Acta Horticuhurae Sin..

[29] Xi W.P., Lu J.F., Qun J.P., Jiao B.N. (2017). Characterization of phenolic profile and antioxidant capacity of different fruit part from lemon (*Citrus limon* Burm.) cultivars. J. Food Sci. Technol. Mysore.

[30] Zhang Y.M., Sun Y.J., Xi W.P., Shen Y., Qiao L.P., Zhong L.Z., Ye X.Q., Zhou Z.Q. (2014). Phenolic compositions and antioxidant capacities of Chinese wild mandarin (*Citrus reticulata* Blanco) fruits. Food Chem..

[31] Chen X.T., Yuan K., Liu H.L. (2010). Phenolic contents and antioxidant activities in ethanol extracts of *Citrus reticulata* Blanco cv. ougan fruit. J. Food Agric. Environ..

[32] Xi W.P., Zhang Y.M., Sun Y.J., Shen Y., Ye X.Q., Zhou Z.Q. (2014). Phenolic composition of Chinese wild mandarin (*Citrus reticulata* Balnco.) pulps and their antioxidant properties. Ind. Crop. Prod..

[33] Schmidt S., Zietz M., Schreiner M., Rohn S., Kroh L.W., Krumbein A. (2010). Genotypic and climatic influences on the concentration and composition of flavonoids in kale (*Brassica oleracea* var. sabellica). Food Chem..

[34] Gil-Izquierdo A., Riquelme M.T., Porras I., Ferreres F. (2004). Effect of the rootstock and interstock grafted in lemon tree (*Citrus limon* (L.) Burm.) on the flavonoid content of lemon juice. J. Agric. Food Chem..

[35] Cheng S.Y., Xu F., Wang Y. (2010). Advances in the study of flavonoids in *Ginkgo biloba* leaves. J. Med. Plants Res..

[36] Khan M.K., Huma Z.E., Dangles O. (2014). A comprehensive review on flavanones, the major citrus polyphenols. J. Food Compos. Anal..

[37] Lu Y., Zhang C., Bucheli P., Wei D. (2006). Citrus flavonoids in fruit and traditional Chinese medicinal food ingredients in China. Plant Foods Hum. Nutr..

[38] Li H., Yang X., Zhu L., Yi H., Chai L., Deng X. (2015). Parentage analysis of natural citrus hybrid ‘Zhelong Zhoupigan’ based on nuclear and chloroplast SSR markers. Acta Horticuhurae Sin..

[39] Dugrand-Judek A., Olry A., Hehn A., Costantino G., Ollitrault P., Froelicher Y., Bourgaud F. (2015). The distribution of coumarins and furanocoumarins in *Citrus* species closely matches citrus phylogeny and reflects the organization of biosynthetic pathways. PLoS ONE.

[40] Xi W., Zhang G., Jiang D., Zhou Z. (2015). Phenolic compositions and antioxidant activities of grapefruit (*Citrus paradisi* Macfadyen) varieties cultivated in China. Int. J. Food Sci. Nutr..

[41] Lou S.N., Ho C.T. (2017). Phenolic compounds and biological activities of small-size citrus: Kumquat and calamondin. J. Food Drug Anal..

[42] Du Q.Z., Chen H. (2010). The methoxyflavones in *Citrus reticulata* Blanco cv. ponkan and their antiproliferative activity against cancer cells. Food Chem..

[43] Xing T.T., Zhao X.J., Zhang Y.D., Li Y.F. (2017). Fast separation and sensitive quantitation of polymethoxylated flavonoids in the peels of *Citrus* using UPLC-Q-TOF-MS. J. Agric. Food Chem..

[44] Sun Y.J., Qiao L.P., Shen Y., Jiang P., Chen J.C., Ye X.Q. (2013). Phytochemical profile and antioxidant activity of physiological drop of *Citrus* fruits. J. Food Sci..

[45] Mishra K., Ojha H., Chaudhury N.K. (2012). Estimation of antiradical properties of antioxidants using DPPH assay: A critical review and results. Food Chem..

[46] Raquel P., Laura B., Fulgencio S.C. (2000). Antioxidant activity of dietary polyphenols as determined by a modified ferric reducing antioxidant power assay. J. Agric. Food Chem..

[47] Nenadis N., Wang L.F., Tsimidou M., Zhang H.Y. (2004). Estimation of scavenging activity of phenolic compounds using the ABTS(+) assay. J. Agric. Food Chem..

[48] Nogata Y., Sakamoto K., Ishii T., Yano M., Shiratsuchi H., Ohta H. (2006). Flavonoid composition of fruit tissues of citrus species. Biosci. Biotechnol. Biochem..

[49] Singleton V.L., Orthofer R., Lamuela Raverntos R.M. (1999). Analysis of total phenols and other oxidation substrates and antioxidants by means of folin-ciocalteu reagent. Methods Enzymol..

[50] Kim D. (2003). Antioxidant capacity of phenolic phytochemicals from various cultivars of plums. Food Chem..

[51] Barreca D., Bellocco E., Caristi C., Leuzzi U., Gattuso G. (2011). Elucidation of the flavonoid and furocoumarin composition and radical-scavenging activity of green and ripe chinotto (*Citrus myrtifolia* Raf.) fruit tissues, leaves and seeds. Food Chem..

[52] Seeram N.P., Aviram M., Zhang Y.J., Henning S.M., Feng L., Dreher M., Heber D. (2008). Comparison of antioxidant potency of commonly consumed polyphenol-rich beverages in the United States. J. Agric. Food Chem..

