# Supplementary material for: Profiling of Flavonoid and Antioxidant Activity of Fruit Tissues from 27 Chinese Local Citrus Cultivars
Source: Plants (Basel). 2020 Feb 5;9(2):196. doi: 10.3390/plants9020196 (PMC7076682; doi:10.3390/plants9020196)
Supplement: Supplementary file 1 [file plants-09-00196-s001.zip › Supplementary material 1.doc]

**Figure S1.** The UV–vis spectrum of the 18 flavonoids tested in this study.

Note: 3.13 min, eriocitrin; 3.41 min, narirutin; 3.51 min, naringin; 3.62 min, hesperidin; 3.70 min, neohesperidin; 3.78, diosmin; 4.23 min, didymin; 5.33 min, isosinensetin; 5.48 min, sinensetin; 5.63 min, 5,7,3′,4′-Tetrathoxyflavone; 5.79 min, 5,6,7,4′- Tetrathoxyflavone; 5.92 min, nobiletin; 6.06 min, 3,5,6,7,8,3′,4′- hetamethoxyflavone; 6.15 min, 5,7,4′-Trimethoxyflavone; 6.29 min, 5-Hydroxy-6,7,8,3′,4′-pentamethoxyflavone; 6.34 min, tangeretin. 6.46, Gardenin A; 6.71 min, Gardenin B.

**Figure S2.** Characteristic UPLC-PDA chromatogram of JJSC peel

**Figure S3.** Characteristic UPLC-PDA chromatogram of RAJD peel

**Figure S4.** Characteristic UPLC-PDA chromatogram of GLQ peel

**Figure S5.** Characteristic UPLC-PDA chromatogram of LPY peel

**Figure S6.** Characteristic UPLC-PDA chromatogram of DJBY peel

**Figure S7.** Characteristic UPLC-PDA chromatogram of GXY peel

**Figure S8.** Characteristic UPLC-PDA chromatogram of TNM peel

**Figure S9.** Characteristic UPLC-PDA chromatogram of LM peel

**Figure S10.** Characteristic UPLC-PDA chromatogram of MLXY peel

**Figure S11.** Characteristic UPLC-PDA chromatogram of JY peel

**Figure S12.** Characteristic UPLC-PDA chromatogram of YCC peel

**Figure S13.** Characteristic UPLC-PDA chromatogram of BDZ peel

**Figure S14.** Characteristic UPLC-PDA chromatogram of NF peel

**Figure S15.** Characteristic UPLC-PDA chromatogram of GSG peel

**Figure S16.** Characteristic UPLC-PDA chromatogram of JG peel

**Figure S17.** Characteristic UPLC-PDA chromatogram of AJHJ peel

**Figure S18.** Characteristic UPLC-PDA chromatogram of ZPG peel

**Figure S19.** Characteristic UPLC-PDA chromatogram of NJ peel

**Figure S20.** Characteristic UPLC-PDA chromatogram of SYJ peel

**Figure S21.** Characteristic UPLC-PDA chromatogram of DHP peel

**Figure S22.** Characteristic UPLC-PDA chromatogram of GLC peel

**Figure S23.** Characteristic UPLC-PDA chromatogram of S26 peel

**Figure S24.** Characteristic UPLC-PDA chromatogram of TC peel

**Figure S25.** Characteristic UPLC-PDA chromatogram of BTC peel

**Figure S26.** Characteristic UPLC-PDA chromatogram of MHQC peel

**Figure S27.** Characteristic UPLC-PDA chromatogram of JJSC pulp

**Figure S28.** Characteristic UPLC-PDA chromatogram of RAJD pulp

**Figure S29.** Characteristic UPLC-PDA chromatogram of GLQ pulp

**Figure S30.** Characteristic UPLC-PDA chromatogram of LPY pulp

**Figure S31.** Characteristic UPLC-PDA chromatogram of DJBY pulp

**Figure S32.** Characteristic UPLC-PDA chromatogram of GXY pulp

**Figure S33.** Characteristic UPLC-PDA chromatogram of TNM pulp

**Figure S34.** Characteristic UPLC-PDA chromatogram of LM pulp

**Figure S35.** Characteristic UPLC-PDA chromatogram of MLXY pulp

**Figure S36.** Characteristic UPLC-PDA chromatogram of JY pulp

**Figure S37.** Characteristic UPLC-PDA chromatogram of YCC pulp

**Figure S38.** Characteristic UPLC-PDA chromatogram of BDZ pulp

**Figure S39.** Characteristic UPLC-PDA chromatogram of NF pulp

**Figure S40.** Characteristic UPLC-PDA chromatogram of GSG pulp

**Figure S41.** Characteristic UPLC-PDA chromatogram of JG pulp

**Figure S42.** Characteristic UPLC-PDA chromatogram of BJ pulp

**Figure S43.** Characteristic UPLC-PDA chromatogram of AJHJ pulp

**Figure S44.** Characteristic UPLC-PDA chromatogram of ZPG pulp

**Figure S45.** Characteristic UPLC-PDA chromatogram of NJ pulp

**Figure S46.** Characteristic UPLC-PDA chromatogram of SYJ pulp

**Figure S47.** Characteristic UPLC-PDA chromatogram of DHP pulp

**Figure S48.** Characteristic UPLC-PDA chromatogram of GLC pulp

**Figure S49.** Characteristic UPLC-PDA chromatogram of S26 pulp

**Figure S50.** Characteristic UPLC-PDA chromatogram of TC pulp

**Figure S51.** Characteristic UPLC-PDA chromatogram of BTC pulp

**Figure S52.** Characteristic UPLC-PDA chromatogram of MHQC pulp

**Figure S53.** Characteristic UPLC-PDA chromatogram of JJSC juice

**Figure S54.** Characteristic UPLC-PDA chromatogram of RAJD juice

**Figure S55.** Characteristic UPLC-PDA chromatogram of GLQ juice

**Figure S56.** Characteristic UPLC-PDA chromatogram of LPY juice

**Figure S57.** Characteristic UPLC-PDA chromatogram of DJBY juice

**Figure S58.** Characteristic UPLC-PDA chromatogram of GXY juice

**Figure S59.** Characteristic UPLC-PDA chromatogram of TNM juice

**Figure S60.** Characteristic UPLC-PDA chromatogram of LM juice

**Figure S61.** Characteristic UPLC-PDA chromatogram of MLXY juice

**Figure S62.** Characteristic UPLC-PDA chromatogram of BDZ juice

**Figure S63.** Characteristic UPLC-PDA chromatogram of NF juice

**Figure S64.** Characteristic UPLC-PDA chromatogram of GSG juice

**Figure S65.** Characteristic UPLC-PDA chromatogram of JG juice

**Figure S66.** Characteristic UPLC-PDA chromatogram of BJ juice

**Figure S67.** Characteristic UPLC-PDA chromatogram of AJHJ juice

**Figure S68.** Characteristic UPLC-PDA chromatogram of ZPG juice

**Figure S69.** Characteristic UPLC-PDA chromatogram of NJ juice

**Figure S70.** Characteristic UPLC-PDA chromatogram of SYJ juice

**Figure S71.** Characteristic UPLC-PDA chromatogram of DHP juice

**Figure S72.** Characteristic UPLC-PDA chromatogram of GLC juice

**Figure S73.** Characteristic UPLC-PDA chromatogram of S26 juice

**Figure S74.** Characteristic UPLC-PDA chromatogram of TC juice

**Figure S75.** Characteristic UPLC-PDA chromatogram of BTC juice

**Figure S76.** Characteristic UPLC-PDA chromatogram of MHQC juice

**Figure S77**. Variations pattern of flavonoids components and contents in the pulp extracts of the 27 local cultivars analyzed in this study.

**Figure S78.** Variations pattern of flavonoids components and contents in the juice extracts of the 27 local cultivars analyzed in this study.
