# Supplementary material for: Profiling of Flavonoid and Antioxidant Activity of Fruit Tissues from 27 Chinese Local Citrus Cultivars
Source: Plants (Basel). 2020 Feb 5;9(2):196. doi: 10.3390/plants9020196 (PMC7076682; doi:10.3390/plants9020196)
Supplement: Supplementary file 1 [file plants-09-00196-s001.zip › Supplementary material 2.doc]

**Table S1.** the characterized component in citrus peels from eight citrus species.

| **Compounds** | ***Citrus grandis* ^a^** | ***Citrus reticulata* ^a^** | ***Citrus sinensis* ^a^** | ***Citrus medica* ^a^** | ***Citrus limonia* ^a^** | ***Citrus ichangensis* ^b^** | ***Citrus aurantium* ^b^** | ***Fortunella crassifolia* ^b^** |
| --- | --- | --- | --- | --- | --- | --- | --- | --- |
| Eriocitrin | 0.185 | 0.444 | 0.327 | 0.353 | 0.794 | 0.344 | 0.000 | 0.520 |
| Narirutin | 0.023 | 0.447 | 1.228 | 0.307 | 0.081 | 0.021 | 0.187 | 0.021 |
| Naringin | 10.229 | 0.184 | 0.061 | 0.030 | 0.310 | 4.377 | 8.793 | 0.152 |
| Hesperidin | 0.152 | 14.363 | 15.144 | 1.628 | 4.713 | 0.043 | 0.359 | 0.062 |
| Neo-hesperidin | 0.511 | 0.678 | 0.000 | 0.000 | 0.000 | 0.224 | 16.208 | 0.182 |
| Diosmin | 0.356 | 0.194 | 0.083 | 0.246 | 0.312 | 0.117 | 0.000 | 1.144 |
| Didymin | 0.003 | 0.044 | 0.202 | 0.426 | 0.015 | 0.016 | 0.000 | 0.000 |
| Isosinensetin | 0.065 | 0.435 | 0.039 | 0.016 | 0.059 | 0.000 | 0.147 | 0.000 |
| Sinensetin | 0.000 | 0.724 | 0.373 | 0.000 | 0.041 | 0.000 | 0.000 | 0.000 |
| 5,7,3',4'-tetramethoxyflavone | 0.000 | 0.048 | 0.127 | 0.000 | 0.000 | 0.178 | 0.000 | 0.000 |
| 5,6,7,4'-tetramethoxyflavone | 0.102 | 0.373 | 0.099 | 0.042 | 0.116 | 0.000 | 0.090 | 0.000 |
| Nobiletin | 0.000 | 5.623 | 0.667 | 0.000 | 0.936 | 0.000 | 0.083 | 0.000 |
| 3,5,6,7,8,3',4'-heptamethoxyflavone | 0.000 | 0.300 | 0.245 | 0.000 | 0.154 | 0.000 | 0.161 | 0.000 |
| 5,7,4'-trimethoxyflavone | 0.374 | 0.430 | 0.000 | 0.000 | 0.474 | 0.333 | 0.574 | 0.000 |
| 5-OH-6,7,8,3',4'-Pentamethoxyflavone | 0.010 | 0.551 | 0.036 | 0.000 | 0.298 | 0.026 | 0.033 | 0.013 |
| Tangeretin | 0.000 | 1.916 | 0.089 | 0.000 | 0.606 | 0.000 | 0.086 | 0.000 |
| Gardenin A | 0.000 | 0.036 | 0.000 | 0.000 | 0.000 | 0.000 | 0.000 | 0.000 |
| Gardenin B | 0.000 | 0.032 | 0.056 | 0.000 | 0.000 | 0.000 | 0.000 | 0.000 |

**^a^** **Statistic mean obtained by PCA standardizing; ^b^ Content of flavonoids obtained by UPLC-PDA; Statistic mean or content of flavonoids exceeding 1 was considered as the characteristic flavonoid, and they were marked by red color.**

**Table S2. Flavanones and diosmin (mg/100g DW) in the peels of 27 citrus cultivars**

| **NO.** | **Cultivars** | **Flavanones** | | | | | | |  | **Flavone** |  |
| --- | --- | --- | --- | --- | --- | --- | --- | --- | --- | --- | --- |
|  |  | **Eriocitrin** | **Narirutin** | **Naringin** | **Hesperidin** | **Neo-hesperidin** | **Didymin** | **Total flavanones** |  | **Diosmin** |  |
| 1 | JJSC | - | 18.72 ± 3.87^k^ | 879.33 ± 82.78^c^ | 35.88 ± 3.67^k^ | 1620.77 ± 172.00^a^ | - | 2554.70 |  | - |  |
| 2 | RAJD | 51.95 ± 3.47^c^ | 2.05 ± 0.01^n^ | 15.18 ± 0.55^h^ | 6.21 ± 1.36^k^ | 18.18 ± 0.18^c^ | - | 93.57 |  | 114.36 ± 6.27^b^ |  |
| 3 | GLQ | 22.12 ± 1.74^hijk^ | 2.72 ± 0.60^n^ | 946.44 ± 72.21^b^ | 11.85 ± 1.51^k^ | 60.67 ± 4.82^c^ | - | 1043.80 |  | - |  |
| 4 | LPY | 20.42 ± 0.33^ijk^ | - | 774.74 ± 62.55^d^ | 8.17 ± 0.91^k^ | 28.42 ± 0.85^c^ | - | 831.75 |  | 6.02 ± 1.29^lmn^ |  |
| 5 | DJBY | - | 6.37 ± 0.60^mn^ | 694.15 ± 48.89^e^ | 7.39 ± 1.97^k^ | 42.39 ± 0.95^c^ | 1.04 ± 0.06^hi^ | 751.34 |  | 14.12 ± 3.41^ghij^ |  |
| 6 | GXY | 31.49 ± 1.63^fghi^ | - | 1676.31 ± 52.76^a^ | 33.39 ± 1.47^k^ | 73.04 ± 3.76^c^ | - | 1814.23 |  | 122.43 ± 8.14^a^ |  |
| 7 | TNM | 83.20 ± 3.90^b^ | 4.69 ± 0.53^mn^ | 11.15 ± 0.69^h^ | 407.09 ± 87^ij^ | - | 1.49 ± 0.14^hi^ | 507.62 |  | 43.96 ± 9.87^c^ |  |
| 8 | LM | 75.62 ± 10.64^b^ | 11.60 ± 1.30^lm^ | 50.88 ± 1.33^gh^ | 535.47 ± 65.71^i^ | - | 1.47 ± 0.14^hi^ | 675.04 |  | 18.52 ± 3.06^fgh^ |  |
| 9 | MLXY | 42.74 ± 3.48^cdef^ | 59.26 ± 8.34^f^ | 5.95 ± 0.01^h^ | 325.52 ± 50.68^j^ | - | 85.25 ± 12.27^a^ | 518.72 |  | 33.38 ± 5.20^de^ |  |
| 10 | JY | 27.79 ± 8.52^ghij^ | 2.05 ± 0.01^n^ | - | - | - | - | 29.84 |  | 15.72 ± 0.78^fghi^ |  |
| 11 | YCC | 34.38 ± 1.25^efgh^ | 2.05 ± 0.01^n^ | 437.67 ± 60.46^f^ | 4.27 ± 0.82^k^ | 22.37 ± 0.95^c^ | 1.57 ± 0.10^hi^ | 502.31 |  | 11.69 ± 0.89^ijkl^ |  |
| 12 | BDZ | 16.96 ± 2.17^jk^ | 18.90 ± 3.62^k^ | 10.94 ± 1.05^h^ | 1292.76 ± 76.24^e^ | - | 3.35 ± 0.21^ghi^ | 1342.91 |  | 30.44 ± 2.00^e^ |  |
| 13 | NF | 23.31 ± 0.77^hij^ | 28.66 ± 0.33^j^ | 26.44 ± 0.46gh | 1779.46 ± 63.03^bc^ | - | 4.93 ± 0.16^gh^ | 1862.80 |  | 33.66 ± 2.74^de^ |  |
| 14 | GSG | 268.69 ± 27.83^a^ | 40.50 ± 1.48^hi^ | 7.96 ± 0.24^h^ | 698.91 ± 14.81^h^ | - | 5.51 ± 0.35^gh^ | 1021.57 |  | 27.76 ± 0.69^e^ |  |
| 15 | JG | 10.32 ± 1.83^kl^ | 145.56 ± 7.24^c^ | 8.86 ± 0.17^h^ | 1780.55 ± 79.99^bc^ | - | 2.80 ± 0.09^hi^ | 1948.09 |  | 21.80 ± 1.43^f^ |  |
| 16 | BJ | 27.29 ± 3.71^ghij^ | 16.21 ± 1.01^kl^ | 14.06 ± 4.53^h^ | 1886.20 ± 198.77^b^ | - | 3.43 ± 0.10^ghi^ | 1947.19 |  | 38.73 ± 4.22^cd^ |  |
| 17 | AJHJ | 20.83 ± 2.51^ijk^ | 47.87 ± 1.59^g^ | 11.76 ± 1.22^h^ | 1738.18 ± 242.10^bc^ | - | 5.64 ± 0.49^gh^ | 1824.28 |  | 15.86 ± 1.89^fghi^ |  |
| 18 | ZPG | 49.89 ± 1.19^cd^ | 18.03 ± 1.00^kl^ | 77.99 ± 0.50^g^ | 39.98 ± 0.60^k^ | 745.25 ± 9.29^b^ | 1.29 ± 0.01^hi^ | 932.43 |  | - |  |
| 19 | NJ | - | 34.66 ± 1.97^ij^ | 6.31 ± 0.62^h^ | 1145.49 ± 94.74^ef^ | - | 1.12 ± 0.04^hi^ | 1187.58 |  | 9.58 ± 1.07^ijklm^ |  |
| 20 | SYJ | 45.87 ± 5.98^cde^ | 59.31 ± 4.08^f^ | 8.63 ± 0.21^h^ | 1687.96 ± 211.41^c^ | - | 7.75 ± 0.41^fg^ | 1809.52 |  | 3.29 ± 2.42^mn^ |  |
| 21 | YXWL | - | 50.55 ± 1.86^g^ | 19.82 ± 0.33^h^ | 1896.73 ± 78.46^b^ | - | 7.77 ± 0.21^fg^ | 1974.87 |  | 20.00 ± 2.02^fg^ |  |
| 22 | DHP | 25.08 ± 2.75^hij^ | 31.39 ± 2.85^j^ | 9.56 ± 0.15^h^ | 1853.29 ± 163.05^bc^ | - | 4.58 ± 0.26^ghi^ | 1923.90 |  | 12.74 ± 0.48^hijk^ |  |
| 23 | GLC | - | 76.89 ± 1.89^e^ | 5.95 ± 0.01^h^ | 960.15 ± 31.68^g^ | - | 12.94 ± 0.24^e^ | 1055.93 |  | 6.81 ± 0.47^klm^ |  |
| 24 | S26 | 34.28 ± 1.46^efgh^ | 43.94 ± 3.44^gh^ | 12.72 ± 1.13^h^ | 1028.14 ± 82.87^fg^ | - | 10.72 ± 0.99^ef^ | 1129.80 |  | 15.80 ± 2.17^fghi^ |  |
| 25 | TC | 38.67 ± 8.92^defg^ | 118.90 ± 4.45^d^ | - | 1487.14 ± 55.13^d^ | - | 18.88 ± 0.67^d^ | 1663.59 |  | 6.49 ± 0.47^lm^ |  |
| 26 | BTC | 45.71 ± 2.67^cde^ | 164.58 ± 14.17^b^ | 6.12 ± 0.15^h^ | 2298.99 ± 190.70^a^ | - | 24.94 ± 2.12^c^ | 2540.34 |  | 6.66 ± 0.53^klm^ |  |
| 27 | MHQC | 44.85 ± 5.23^cde^ | 209.64 ± 1.58^a^ | 5.95 ± 0.02^h^ | 1797.36 ± 6.51^bc^ | - | 33.38 ± 0.19^b^ | 2091.18 |  | 5.65 ± 0.18^lmn^ |  |

Data are expressed as means ± SD. The different small letters after the means represent significance in the same tissue of citrus at 0.05 level. -: not detected.

**Table** **S3. Flavonoids (mg/100g DW) in the pulps of 27 citrus cultivars**

| **NO.** | **Cultivars** | **Flavanones** | | | | | | |  | **Flavone** |  | **PMFs** | |
| --- | --- | --- | --- | --- | --- | --- | --- | --- | --- | --- | --- | --- | --- |
|  |  | **Eriocitrin** | **Narirutin** | **Naringin** | **Hesperidin** | **Neo-hesperidin** | **Didymin** | **Total flavanones** |  | **Diosmin** |  | **5,7,3',4'-tetramethoxyflavone** | **Tangeretin** |
| 1 | JJSC | - | 19.17 ± 0.89^jk^ | 326.44 ± 7.46^c^ | 53.05 ± 0.29^i^ | 517.10 ± 7.75^a^ | 0.81 ± 0.03^ij^ | 916.58 |  | 189.16 ± 2.79^a^ |  | - | - |
| 2 | RAJD | 26.73 ± 1.16^c^ | - | 10.33 ± 0.21^h^ | 5.07 ± 0.33^l^ | - | - | 42.13 |  | 83.97 ± 7.40^b^ |  | 4.67 ± 0.04 | - |
| 3 | GLQ | 19.77 ± 1.48^def^ | - | 777.65 ± 17.75^a^ | 21.71 ± 2.06^jkl^ | 28.26 ± 0.65^e^ | - | 847.39 |  | 25.52 ± 2.42^e^ |  | - | 2.57 ± 0.06^b^ |
| 4 | LPY | - | - | 287.78 ± 16.99^d^ | 2.73 ± 0.63^l^ | 47.88 ± 1.01^cd^ | - | 338.38 |  | 67.98 ± 3.78^c^ |  | - | - |
| 5 | DJBY | - | - | 199.55 ± 2.89^f^ | 5.98 ± 0.57^l^ | 34.29 ± 1.73^de^ | 1.07 ± 0.03^ij^ | 240.9 |  | 38.47 ± 3.75^d^ |  | - | - |
| 6 | GXY | 15.95 ± 1.32^ef^ | - | 616.27 ± 16.2^b^ | 13.00 ± 2.27^kl^ | 52.20 ± 1.20^c^ | - | 697.41 |  | 62.6 ± 25.64^c^ |  | - | - |
| 7 | TNM | 16.75 ± 0.68^ef^ | - | 9.55 ± 0.31^h^ | 36.23 ± 5.20^ijk^ | 19.51 ± 0.25^e^ | - | 82.04 |  | - |  | - | - |
| 8 | LM | 66.72 ± 4.20^a^ | - | 6.78 ± 0.68^h^ | 46.45 ± 5.46^ij^ | 18.70 ± 0.08^e^ | 1.28 ± 0.07^ij^ | 139.93 |  | - |  | - | - |
| 9 | MLXY | 24.11 ± 4.43^cd^ | 26.85 ± 3.79^hi^ | - | 136.39 ± 19.45^g^ | 22.96 ± 0.49^e^ | 34.3 ± 6.43^a^ | 244.61 |  | 17.36 ± 3.27^e^ |  | - | - |
| 10 | JY | 23.76 ± 2.93^cd^ | - | 10.63 ± 0.20^h^ | 18.55 ± 1.76^jkl^ | - | - | 52.94 |  | 21.64 ± 1.59^e^ |  | - | - |
| 11 | YCC | 19.30 ± 0.45^def^ | - | 245.53 ± 15.07^e^ | 22.47 ± 2.00^jkl^ | - | - | 287.30 |  | - |  | - | 1.59 ± 0.09^f^ |
| 12 | BDZ | - | 23.91 ± 2.40^ij^ | - | 228.56 ± 14.79^de^ | - | 2.24 ± 0.22^ghij^ | 254.72 |  | - |  | - | 1.85 ± 0.07^d^ |
| 13 | NF | - | 25.22 ± 3.06^hi^ | - | 237.36 ± 17.00^d^ | - | 2.56 ± 0.45^fghij^ | 265.14 |  | - |  | - | 1.82 ± 0.05^d^ |
| 14 | GSG | 45.50 ± 1.65^b^ | 33.18 ± 1.13^g^ | 8.02 ± 0.89^h^ | 162.34 ± 7.03^fg^ | 19.86 ± 0.08^e^ | 4.17 ± 0.37^defgh^ | 273.06 |  | - |  | - | 1.81 ± 0.09^d^ |
| 15 | JG | 23.92 ± 0.94^cd^ | 120.30 ± 5.49^b^ | - | 386.18 ± 24.78^b^ | - | 1.01 ± 0.04^ij^ | 531.42 |  | - |  | - | - |
| 16 | BJ | - | 32.14 ± 1.46^g^ | - | 415.59 ± 49.59^a^ | - | 5.60 ± 0.70^d^ | 453.32 |  | - |  | - | 1.68 ± 0.08^e^ |
| 17 | AJHJ | - | 29.60 ± 3.92^gh^ | 7.68 ± 0.11^h^ | 168.55 ± 3.10^f^ | - | 1.51 ± 0.82^ij^ | 207.33 |  | - |  | - | 2.62 ± 0.14^b^ |
| 18 | ZPG | 45.24 ± 4.25^b^ | 26.09 ± 2.08^hi^ | 160.03 ± 1.67^g^ | 13.85 ± 1.40^kl^ | 287.49 ± 13.61^b^ | - | 532.70 |  | - |  | - | 2.24 ± 0.09^c^ |
| 19 | NJ | - | 30.05 ± 3.28^gh^ | - | 161.24 ± 1.39^fg^ | - | 4.64 ± 1.03^defg^ | 195.93 |  | - |  | - | 2.64 ± 0.04^b^ |
| 20 | SYJ | - | 39.16 ± 1.28^f^ | - | 144.18 ± 4.24^fg^ | - | 2.73 ± 0.10^efghi^ | 186.07 |  | - |  | - | - |
| 21 | YXWL | - | 41.26 ± 1.13^ef^ | - | 204.74 ± 11.87^e^ | - | 5.12 ± 0.55^de^ | 251.12 |  | - |  | - | 3.84 ± 0.09^a^ |
| 22 | DHP | - | 18.19 ± 0.29^k^ | - | 96.33 ± 3.58^h^ | - | 1.94 ± 0.13^hij^ | 116.46 |  | - |  | - | 1.86 ± 0.09^d^ |
| 23 | GLC | 24.55 ± 2.07^cd^ | 58.63 ± 7.08^d^ | - | 231.10 ± 20.90^de^ | - | 5.40 ± 0.88^d^ | 319.67 |  | - |  | - | - |
| 24 | S26 | 21.51 ± 1.44^cde^ | 45.09 ± 1.52^e^ | - | 218.82 ± 3.40^de^ | - | 4.78 ± 0.07^def^ | 290.20 |  | - |  | - | - |
| 25 | TC | - | 41.62 ± 5.91^ef^ | - | 103.14 ± 1.90^h^ | - | 4.19 ± 0.69^defgh^ | 148.95 |  | - |  | - | - |
| 26 | BTC | 13.85 ± 0.93^f^ | 69.67 ± 5.56^c^ | - | 385.37 ± 40.82^b^ | - | 10.18 ± 1.36^c^ | 479.07 |  | - |  | - | - |
| 27 | MHQC | 24.45 ± 3.69^cd^ | 153.48 ± 4.14^a^ | - | 345.79 ± 14.98^c^ | - | 13.15 ± 0.43^b^ | 536.87 |  | - |  | - | - |

Data are expressed as means ± SD. The different small letters after the means represent significance in the same tissue of citrus at 0.05 level. -: not detected.

**Table S4. Flavonoids (μg/mL FW) in the juices of 27 citrus cultivars**

| **NO.** | **Cultivars** | **Flavanones** | | | | | | |  | **Flavone** |  | **PMFs** |  |
| --- | --- | --- | --- | --- | --- | --- | --- | --- | --- | --- | --- | --- | --- |
|  |  | **Eriocitrin** | **Narirutin** | **Naringin** | **Hesperidin** | **Neo-hesperidin** | **Didymin** | **Total flavanones** |  | **Diosmin** |  | **Tangeretin** |  |
| 1 | JJSC | - | - | 79.19 ± 1.18^c^ | 4.68 ± 0.28^j^ | 144.85 ± 1.61^a^ | - | 323.73 |  | - |  | 3.34 ± 0.07^def^ |  |
| 2 | RAJD | - | - | - | 0.58 ± 0.02^j^ | - | - | 439.25 |  | 46.77 ± 2.47^a^ |  | 3.46 ± 0.04^cdef^ |  |
| 3 | GLQ | - | - | 419.28 ± 19.68^a^ | 8.85 ± 1.37^j^ | - | - | 84.83 |  | 7.83 ± 1.48^d^ |  | 3.29 ± 0.12^ef^ |  |
| 4 | LPY | - | - | 181.28 ± 2.95^b^ | 12.17 ± 0.25^j^ | 42.54 ± 1.78^c^ | - | 154.35 |  | 21.38 ± 2.92^b^ |  | 3.09 ± 0.01^f^ |  |
| 5 | DJBY | - | - | 40.82 ± 0.52^d^ | 3.14 ± 0.22^j^ | 37.52 ± 0.56^d^ | 1.80 ± 0.05^h^ | 132.62 |  | - |  | 3.42 ± 0.27^cdef^ |  |
| 6 | GXY | - | - | 74.57 ± 1.51^c^ |  | 39.53 ± 1.13^d^ | - | 153.67 |  | 14.79 ± 1.97^c^ |  | 3.64 ± 0.55^cdef^ |  |
| 7 | TNM | - | - | - | 34.57 ± 3.10^i^ | - | - | 115.88 |  | - |  | 3.32 ± 0.07^ef^ |  |
| 8 | LM | 24.06 ± 0.49^j^ | 24.06 ± 0.49^j^ | - | 34.06 ± 0.59^i^ | - | 2.36 ± 0.06^g^ | 49.18 |  | - |  | 3.70 ± 0.34^bcde^ |  |
| 9 | MLXY | 28.46 ± 0.28^g^ | 28.46 ± 0.28^g^ | - | 42.13 ± 1.86^hi^ | - | 12.50 ± 0.18^a^ | 98.19 |  | - |  | 3.76 ± 0.28^bcde^ |  |
| 12 | BDZ | 27.56 ± 0.07^ghi^ | 27.56 ± 0.07^ghi^ | - | 80.00 ± 3.67^e^ | - | 2.26 ± 0.14^g^ | 100.27 |  | - |  | 4.32 ± 0.31^a^ |  |
| 13 | NF | 30.78 ± 0.60^f^ | 30.78 ± 0.60^f^ | - | 141.68 ± 12.54^c^ | - | 2.53 ± 0.21^fg^ | 69.29 |  | - |  | 3.89 ± 0.28^abcd^ |  |
| 14 | GSG | 28.25 ± 0.51^gh^ | 28.25 ± 0.51^gh^ | - | 50.53 ± 3.20^gh^ | - | 2.46 ± 0.17^fg^ | 228.87 |  | - |  | 3.59 ± 0.10^cdef^ |  |
| 15 | JG | 78.39 ± 1.92^a^ | 78.39 ± 1.92^a^ | - | 199.35 ± 14.90^a^ | - | 10.33 ± 0.98^b^ | 185.09 |  | - |  | 3.69 ± 0.28^cde^ |  |
| 16 | BJ | 28.30 ± 0.88^gh^ | 28.30 ± 0.88^gh^ | - | 66.06 ± 4.12^ef^ | - | 2.47 ± 0.29^fg^ | 129.09 |  | - |  | 3.44 ± 0.11^cdef^ |  |
| 17 | AJHJ | 26.57 ± 0.53^i^ | 26.57 ± 0.53^i^ | - | 66.46 ± 11.84^ef^ | - | 1.73 ± 0.27^h^ | 50.81 |  | - |  | 3.43 ± 0.15^cdef^ |  |
| 18 | ZPG | - | - | 24.81 ± 0.37^e^ | - | 69.19 ± 1.51^b^ | - | 191.67 |  | - |  | 3.49 ± 0.18^cdef^ |  |
| 19 | NJ | 36.63 ± 0.80^d^ | 36.63 ± 0.80^d^ | - | 141.85 ± 1.24^c^ | - | 2.71 ± 0.07^fg^ | 291.76 |  | - |  | 3.9 ± 0.15^abc^ |  |
| 20 | SYJ | 27.86 ± 0.50^gh^ | 27.86 ± 0.50^gh^ | - | 76.85 ± 4.88^e^ | - | 2.89 ± 0.09^ef^ | 87.21 |  | - |  | 4.21 ± 0.41^ab^ |  |
| 21 | YXWL | 29.81 ± 0.34^f^ | 29.81 ± 0.34^f^ | - | 59.98 ± 1.77^fg^ | - | 2.33 ± 0.06^g^ | 95.95 |  | - |  | 3.83 ± 0.30^abcde^ |  |
| 22 | DHP | 27.17 ± 0.46^hi^ | 27.17 ± 0.46^hi^ | - | 99.38 ± 26.70^d^ | - | - | 86.85 |  | - |  | 3.54 ± 0.48^cdef^ |  |
| 23 | GLC | 34.20 ± 0.20^e^ | 34.20 ± 0.20^e^ | - | 81.56 ± 4.92^e^ | - | 3.20 ± 0.14^ef^ | 93.00 |  | - |  | 3.52 ± 0.41^cdef^ |  |
| 24 | S26 | 28.38 ± 0.50^g^ | 28.38 ± 0.50^g^ | - | 34.98 ± 1.12^i^ | - | 2.34 ± 0.10^g^ | 76.80 |  | - |  | 3.59 ± 0.14^cdef^ |  |
| 25 | TC | 41.25 ± 0.70^c^ | 41.2 5± 0.70^c^ | - | 67.38 ± 6.83^ef^ | - | 3.70 ± 0.15^d^ | 28.30 |  | - |  | 3.55 ± 0.26^cdef^ |  |
| 26 | BTC | 46.95 ± 1.37^b^ | 46.95 ± 1.37^b^ | - | 173.11 ± 22.55^b^ | - | 5.21 ± 0.47^c^ | 217.92 |  | - |  | 3.60 ± 0.27^cdef^ |  |
| 27 | MHQC | 46.24 ± 0.58^b^ | 46.24 ± 0.58^b^ | - | 75.39 ± 1.04^ef^ | - | 3.79 ± 0.16^d^ | 130.09 |  | - |  | 3.67 ± 0.43^cde^ |  |

Data are expressed as means ± SD. The different small letters after the means represent significance in the same tissue of citrus at 0.05 level. -: not detected.

**Table S5. PMFs (mg/100g DW) in the peels of 27 citrus cultivars**

| **NO.** | **Cultivars** | **Isosinensetin** | **Sinensetin** | **5,7,3',4'-TMF** | **5,6,7,4'-TMF** | **Nobiletin** | **HMF** | **5,7,4'-TRMF** | **5-OH-PTMF** | **Tangeretin** | **Gardenin A** | **Gardenin B** | **Total PMFs** |
| --- | --- | --- | --- | --- | --- | --- | --- | --- | --- | --- | --- | --- | --- |
| 1 | JJSC | 14.74 ± 0.70^hi^ | - | - | 9.05 ± 0.04^no^ | 8.33 ± 0.73^l^ | 16.14 ± 0.23^j^ | 57.41 ± 0.75^b^ | 3.32 ± 0.10^lm^ | 8.65 ± 0.22^i^ | - | - | 117.63 |
| 2 | RAJD | - | - | - | - | - | - | - | 1.32 ± 0.00^m^ | - | - | - | 1.32 |
| 3 | GLQ | 1.50 ± 0.17^n^ | - | - | 10.54 ± 0.11^kl^ | - | - | 34.00 ± 0.09^k^ | - | - | - | - | 46.05 |
| 4 | LPY | 11.21 ± 0.47^j^ | - | - | 9.15 ± 0.05^mno^ | - | - | 31.57 ± 0.85^m^ | - | - | - | - | 51.93 |
| 5 | DJBY | 9.76 ± 0.87^k^ | - | - | 9.97 ± 0.25^lmn^ | - | - | 32.28 ± 0.10^lm^ | 3.90 ± 0.47^lm^ | - | - | - | 55.92 |
| 6 | GXY | 3.53 ± 0.12^lm^ | - | - | 11.26 ± 0.28^jk^ | - | - | 51.70 ± 0.27^d^ | - | - | - | - | 66.50 |
| 7 | TNM | 3.30 ± 0.25^lm^ | 8.18 ± 0.80^j^ | 17.83±0.45a | 11.91 ± 0.34^j^ | 33.58 ± 2.85^k^ | 5.69 ± 0.02^m^ | 34.03 ± 0.37^k^ | 10.89 ± 0.69^j^ | 89.23 ± 4.44^g^ | - | - | 214.63 |
| 8 | LM | 8.45 ± 0.33^k^ | - | - | 11.33 ± 0.10^jk^ | 153.63 ± 3.02^h^ | 25.18 ± 0.40^fg^ | 60.75 ± 0.48^a^ | 48.70 ± 0.79^e^ | 32.07 ± 0.45^h^ | - | - | 340.10 |
| 9 | MLXY | 3.20 ± 1.88^lm^ | - | - | 8.48 ± 0.50^o^ | - | - | - | - | - | - | - | 11.68 |
| 10 | JY | - | - | - | - | - | - | - | - | - | - | - | - |
| 11 | YCC | - | - | - | - | - | - | 45.42 ± 0.56^g^ | 2.63 ± 0.32^lm^ | - | - | - | 48.06 |
| 12 | BDZ | 14.10 ± 0.66^i^ | 20.32 ± 1.30^h^ | 4.69 ± 0.07^j^ | 21.45 ± 1.01^g^ | 150.35 ± 7.97^h^ | 29.50 ± 1.09^d^ | 40.18 ± 0.15^j^ | 8.46 ± 0.32^k^ | 94.52 ± 4.14^fg^ | - | - | 383.56 |
| 13 | NF | 72.71 ± 0.81^b^ | 67.93 ± 2.57^d^ | 7.23 ± 0.10^h^ | 43.81 ± 0.56^d^ | 693.58 ± 11.05^d^ | 24.13 ± 0.38^gh^ | 43.09 ± 0.14^h^ | 79.74 ± 1.38^b^ | 288.81 ± 4.59^b^ | - | - | 1321.02 |
| 14 | GSG | 104.58 ± 0.94^a^ | 297.99 ± 3.29^a^ | 6.84 ± 0.10^h^ | 105.40 ± 1.15^a^ | 895.91 ± 10.25^b^ | - | 32.68 ± 0.06^l^ | 15.14 ± 0.28^i^ | 133.89 ± 1.59^e^ | - | - | 1592.44 |
| 15 | JG | 45.30 ± 1.29^e^ | 66.32 ± 2.74^d^ | 7.12 ± 0.15^h^ | 16.99 ± 0.17^i^ | 691.23 ± 12.82^d^ | 92.83 ± 1.72^a^ | 52.31 ± 1.50^d^ | 57.68 ± 1.20^d^ | 100.99 ± 1.71^f^ | - | - | 1130.78 |
| 16 | BJ | 15.81 ± 0.86^h^ | 22.26 ± 1.89^h^ | 5.65 ± 0.30^i^ | 18.68 ± 0.60^h^ | 194.28 ± 12.03^g^ | 62.67 ± 3.32^b^ | 54.48 ± 0.92^c^ | 19.35 ± 1.15^h^ | 97.48 ± 5.64^f^ | - | - | 490.67 |
| 17 | AJHJ | 60.04 ± 1.35^d^ | 80.60 ± 1.94^c^ | 8.43 ± 0.17^g^ | 45.08 ± 0.96^c^ | 767.7 5± 18.42^c^ | 27.66 ± 0.92^e^ | 41.94 ± 0.34^i^ | 69.21 ± 2.09^c^ | 222.31 ± 7.78^c^ | - | - | 1323.01 |
| 18 | ZPG | 8.61 ± 0.08^k^ | 13.94 ± 0.28^i^ | - | 11.83 ± 0.10^j^ | 196.98 ± 1.17^g^ | 32.74 ± 0.19^c^ | 32.95 ± 0.20^l^ | 25.84 ± 0.14^g^ | 89.54 ± 0.68^g^ | - | - | 412.43 |
| 19 | NJ | 24.37 ± 0.43^f^ | 27.77 ± 0.89^g^ | - | 21.85 ± 0.31^g^ | 423.42 ± 10.85^e^ | 9.39 ± 0.17^l^ | 32.08 ± 0.07^lm^ | 40.78 ± 0.96^f^ | 139.98 ± 3.60^e^ | - | 11.87 ± 0.02^c^ | 730.40 |
| 20 | SYJ | 19.09 ± 0.45^g^ | 28.84 ± 1.45^g^ | - | 25.60 ± 0.77^f^ | 340.71 ± 13.67^f^ | 10.69 ± 0.31^l^ | 50.56 ± 0.07^e^ | 8.31 ± 0.39^k^ | 177.28 ± 6.99^d^ | - | - | 661.09 |
| 21 | YXWL | 67.43 ± 2.40^c^ | 101.27 ± 5.16^b^ | 5.77 ± 0.10^i^ | 60.21 ± 0.96^b^ | 1137.05 ± 18.52^a^ | 13.54 ± 0.36^k^ | 47.6 ± 0.44^f^ | 211.38 ± 5.27^a^ | 536.75 ± 10.90^a^ | - | - | 2180.99 |
| 22 | DHP | 46.54 ± 0.68^e^ | 69.23 ± 1.07^d^ | 7.11 ± 0.17^h^ | 39.85 ± 0.63^e^ | 693.69 ± 12.14^d^ | 26.60 ± 0.43^ef^ | - | 69.88 ± 0.98^c^ | 226.26 ± 5.90^c^ | 40.03 ± 0.06 | 23.23 ± 0.08^b^ | 1290.12 |
| 23 | GLC | 3.97 ± 0.11^lm^ | 27.48 ± 0.60^g^ | 10.15 ± 0.17^f^ | 9.22 ± 0.01^mno^ | 58.31 ± 0.73^j^ | 22.12 ± 0.89^i^ | - | 3.64 ± 0.07^lm^ | 7.97 ± 0.14^i^ | - | - | 142.85 |
| 24 | S26 | 3.75 ± 0.10^lm^ | 41.65 ± 0.65^f^ | 13.11 ± 0.04^c^ | 9.87 ± 0.07^lmn^ | 77.78 ± 0.52^i^ | 25.09 ± 0.08^fg^ | - | 3.66 ± 0.06^lm^ | 10.24 ± 0.15^i^ | - | - | 185.14 |
| 25 | TC | 4.18 ± 0.14^lm^ | 45.58 ± 1.09^e^ | 16.88 ± 0.19^b^ | 10.04 ± 0.09^lm^ | 72.34 ± 1.98^ij^ | 25.86 ± 0.82^efg^ | - | 2.77 ± 0.07^lm^ | 8.97 ± 0.16^i^ | - | - | 186.62 |
| 26 | BTC | 4.64 ± 0.49^l^ | 40.92 ± 3.57^f^ | 11.99 ± 0.53^d^ | 11.33 ± 0.29^jk^ | 66.10 ± 5.09^ij^ | 27.19 ± 1.72^e^ | - | 4.18 ± 0.25^l^ | 7.35 ± 0.23^i^ | - | - | 173.71 |
| 27 | MHQC | 2.89 ± 0.06^mn^ | 30.81 ± 0.95^g^ | 11.51 ± 0.14^e^ | 9.28 ± 0.03^mno^ | 58.89 ± 1.35^j^ | 22.47 ± 0.32^hi^ | - | 3.58 ± 0.11^lm^ | 10.01 ± 0.27^i^ | - | 27.89 ± 0.08a | 178.18 |

Data are expressed as means ± SD. The different small letters after the means represent significance in the same tissue of citrus at 0.05 level. -: not detected. TMF: tetramethoxyflavone; HMF: 3,5,6,7,8,3',4'-heptamethoxyflavone; PTMF: 6,7,8,3',4'-Pentamethoxyflavone; TRMF: trimethoxyflavone.

**Table S6 Linearity, LOD, LOQ, recoveries and precision of 18 standard flavonoids obtained by UPLC-PDA.**

| **compounds** | **regression equation** | **R^2^** | **linear range (μg/mL)** | **LOD (μg/mL)** | **LOQ (μg/mL)** | **repeatability** | | **Precision** | |
| --- | --- | --- | --- | --- | --- | --- | --- | --- | --- |
|  |  |  |  |  |  | **Recovery (%)** | **RSD (%)** | **Inter-day** | **Intra-day** |
| Eriocitrin | y = 1591.1x - 2112.3 | 0.9998 | 0.4-200 | 0.6367 | 1.5934 | 97.65 | 3.76 | 0.95 | 2.85 |
| Narirutin | y = 8401.5x - 5371.9 | 0.9998 | 0.5-125 | 0.1543 | 0.7268 | 103.21 | 4.75 | 1.62 | 4.13 |
| Naringin | y = 7029x - 5025.6 | 0.9993 | 0.4-200 | 0.243 | 0.4867 | 94.54 | 5.35 | 1.03 | 4.56 |
| Hesperidin | y = 32189x + 2825.7 | 0.9999 | 0.4-200 | 0.25 | 0.5384 | 98.75 | 2.85 | 0.88 | 2.46 |
| Neo-hesperidin | y = 5140.4x - 13791 | 0.9995 | 0.5-500 | 0.434 | 0.835 | 101.27 | 4.85 | 1.39 | 3.46 |
| Diosmin | y = 2375.2x - 242.99 | 0.9999 | 0.4-200 | 0.265 | 0.7324 | 95.32 | 4.62 | 2.12 | 3.44 |
| Didymin | y = 24995x - 3153.2 | 0.9994 | 0.4-200 | 0.224 | 0.765 | 96.72 | 3.23 | 1.35 | 2.98 |
| 5,7,8,3′,4′-Pentamethoxyflavone (Isosinensetin) | y = 7231.4x - 413.56 | 0.9999 | 0.4-200 | 0.39 | 0.9654 | 99.43 | 3.47 | 1.05 | 1.72 |
| 5,6,7,3′,4′-Pentamethoxyflavone (Sinensetin) | y = 9590.9x + 4037.8 | 0.9998 | 0.25-500 | 0.1106 | 0.245 | 100.02 | 6.32 | 1.43 | 3.76 |
| 5,7,3′,4′-Tetramethoxyflavone | y = 8593.4x - 5085.3 | 0.9999 | 0.25-125 | 0.25 | 0.54 | 103.54 | 5.43 | 1.32 | 4.34 |
| 5,6,7,4′-Tetramethoxyflavone | y = 9067.4x - 11468 | 0.9994 | 0.25-500 | 0.24 | 0.45 | 97.43 | 2.95 | 0.74 | 1.68 |
| 5,7,4’-Trimethoxyflavone | y = 4772.7x - 23423 | 0.9992 | 0.5-500 | 0.25 | 0.5847 | 98.56 | 4.12 | 1.87 | 2.88 |
| 5,6,7,8,3′,4′-hexamethoxyflavone (Nobiletin) | y = 8798.1x + 9924.1 | 1 | 0.5-500 | 0.0645 | 0.2014 | 96.74 | 5.78 | 0.79 | 3.12 |
| 3,5,6,7,8,3′,4′-heptamethoxyflavone (Heptamethoxyflavone) | y = 8098.4x - 9866.3 | 0.9999 | 0.25-500 | 0.245 | 0.482 | 102.44 | 2.78 | 1.54 | 4.42 |
| 5,6,7,8,4′-Pentamethoxyflavone (Tangeretin) | y = 10775x - 6784.6 | 0.9999 | 0.25-500 | 0.216 | 0.365 | 105.01 | 3.96 | 1.26 | 2.76 |
| 5-hydroxy-6,7,8,3′,4′-Pentamethoxyflavone | y = 5333.3x - 4702.2 | 1 | 0.25-500 | 0.25 | 0.56 | 98.65 | 5.02 | 0.98 | 4.11 |
| 5-hydroxy-6,7,8,3′,4′,5′-Hexamethoxyflavone (Gardenin A) | y = 2981.3x - 2612 | 0.9999 | 0.5-250 | 0.4586 | 1.3981 | 101.24 | 4.66 | 1.12 | 3.66 |
| 5-hydroxy-6,7,8,4’- Tetramethoxyflavone (5-Demethyltangeretin, Gardenin B) | y = 4067.5x - 4295.5 | 0.9997 | 0.5-500 | 0.5001 | 1.2995 | 99.86 | 1.98 | 0.95 | 3.35 |
